# Supplementary material for: Fast and flexible processing of large FRET image stacks using the FRET-IBRA toolkit
Source: PLoS Comput Biol. 2022 Apr 4;18(4):e1009242. doi: 10.1371/journal.pcbi.1009242 (PMC9009774; doi:10.1371/journal.pcbi.1009242)
Supplement: Attachment — Submitted filename: Tests performed with FRET-IBRA.pdf [file pcbi.1009242.s001.pdf]

Tests performed with FRET-IBRA (July 2021)

Datasets tested:
Four 16-bit datasets of growing Arabidopsis pollen tubes were used to test the pipeline.

1) pH283 - 1024x1024 - 594MB/channel (pHluorin)
2) pH213 - 1024x1024 - 447MB/channel (pHluorin)
3) YC124 - 640x640 - 101MB/channel (Yellow Cameleon)
4) YC170 - 480x480 - 79MB/channel (Yellow Cameleon)

Specs:
macOS Mojave
MacBook Pro 2.5 GHz Intel Core i7
16 GB 1600 MHz DDR3

Log-files:

pH283

```
(base) COMP:~ reviewer$ cd fret-ibra/ibra/
(base) COMP:ibra reviewer$ conda activate py38
(py38) COMP:ibra reviewer$ ./ibra.py -c Config_pH283-0.cfg -a -t -s -v
/opt/anaconda3/envs/py38/lib/python3.8/site-packages/pims/api.py:191: UserWarning:
<class 'pims.tiff_stack.TiffStack_tifffile'> errored: 'NoneType' object has no attribute
'TiffFile'
  warn(message)
Acceptor (Background Subtraction) Frame Number: 1
Traceback (most recent call last):
  File "./ibra.py", line 180, in <module>
    main()
  File "./ibra.py", line 144, in main

bs.background(verbose,logger,work_inp_path,work_out_path,ext,res,module,eps,win,p
arallel,anim_save,h5_save,tiff_save,frange)
  File "/Users/reviewer/fret-ibra/ibra/background_subtraction.py", line 243, in background
    all.stack_workflow(parallel)
  File "/Users/reviewer/fret-ibra/ibra/background_subtraction.py", line 117, in
stack_workflow
    result = fr.frame_workflow()
  File "/Users/reviewer/fret-ibra/ibra/background_subtraction.py", line 209, in
frame_workflow
    self.properties()
  File "/Users/reviewer/fret-ibra/ibra/background_subtraction.py", line 135, in properties
    self.im_tile = block(self.im_frame,super().dim)
  File "/Users/reviewer/fret-ibra/ibra/functions.py", line 270, in block
    return (data.reshape(data.shape[0] // size, size, -1, size)
ValueError: cannot reshape array of size 1048576 into shape (40,25,newaxis,25)
```

pH213

```
(base) COMP:~ reviewer$ conda activate py38
(py38) COMP:~ reviewer$ cd fret-ibra/ibra/
(py38) COMP:ibra reviewer$ ./ibra.py -c Config_pH213-0.cfg -a -t -s -v
/opt/anaconda3/envs/py38/lib/python3.8/site-packages/pims/api.py:191: UserWarning:
<class 'pims.tiff_stack.TiffStack_tifffile'> errored: 'NoneType' object has no attribute
'TiffFile'
  warn(message)
Acceptor (Background Subtraction) Frame Number: 1
Traceback (most recent call last):
  File "./ibra.py", line 180, in <module>
    main()
  File "./ibra.py", line 144, in main

bs.background(verbose,logger,work_inp_path,work_out_path,ext,res,module,eps,win,p
arallel,anim_save,h5_save,tiff_save,frange)
  File "/Users/reviewer/fret-ibra/ibra/background_subtraction.py", line 243, in background
    all.stack_workflow(parallel)
  File "/Users/reviewer/fret-ibra/ibra/background_subtraction.py", line 117, in
stack_workflow
    result = fr.frame_workflow()
  File "/Users/reviewer/fret-ibra/ibra/background_subtraction.py", line 209, in
frame_workflow
    self.properties()
  File "/Users/reviewer/fret-ibra/ibra/background_subtraction.py", line 135, in properties
    self.im_tile = block(self.im_frame,super().dim)
  File "/Users/reviewer/fret-ibra/ibra/functions.py", line 270, in block
    return (data.reshape(data.shape[0] // size, size, -1, size)
ValueError: cannot reshape array of size 1048576 into shape (40,25,newaxis,25)
(py38) COMP:ibra reviewer$
```

YC124

```
(base) COMP:~ reviewer$ conda activate py38
(py38) COMP:~ reviewer$ cd fret-ibra/ibra/
(py38) COMP:ibra reviewer$ ./ibra.py -c Config_YC124-0.cfg -a -t -s -v
/opt/anaconda3/envs/py38/lib/python3.8/site-packages/pims/api.py:191: UserWarning:
<class 'pims.tiff_stack.TiffStack_tifffile'> errored: 'NoneType' object has no attribute
'TiffFile'
  warn(message)
Acceptor (Background Subtraction) Frame Number: 1
Acceptor (Background Subtraction) Frame Number: 2
Acceptor (Background Subtraction) Frame Number: 3
Acceptor (Background Subtraction) Frame Number: 4
Acceptor (Background Subtraction) Frame Number: 5
```

Acceptor (Background Subtraction) Frame Number: 6
Acceptor (Background Subtraction) Frame Number: 7
Acceptor (Background Subtraction) Frame Number: 8
Acceptor (Background Subtraction) Frame Number: 9
Acceptor (Background Subtraction) Frame Number: 10
Acceptor (Background Subtraction) Frame Number: 11
Acceptor (Background Subtraction) Frame Number: 12
Acceptor (Background Subtraction) Frame Number: 13
Acceptor (Background Subtraction) Frame Number: 14
Acceptor (Background Subtraction) Frame Number: 15
Acceptor (Background Subtraction) Frame Number: 16
Acceptor (Background Subtraction) Frame Number: 17
Acceptor (Background Subtraction) Frame Number: 18
Acceptor (Background Subtraction) Frame Number: 19
Acceptor (Background Subtraction) Frame Number: 20
Acceptor (Background Subtraction) Frame Number: 21
Acceptor (Background Subtraction) Frame Number: 22
Acceptor (Background Subtraction) Frame Number: 23
Acceptor (Background Subtraction) Frame Number: 24
Acceptor (Background Subtraction) Frame Number: 25
Acceptor (Background Subtraction) Frame Number: 26
Acceptor (Background Subtraction) Frame Number: 27
Acceptor (Background Subtraction) Frame Number: 28
Acceptor (Background Subtraction) Frame Number: 29
Acceptor (Background Subtraction) Frame Number: 30
Acceptor (Background Subtraction) Frame Number: 31
Acceptor (Background Subtraction) Frame Number: 32
Acceptor (Background Subtraction) Frame Number: 33
Acceptor (Background Subtraction) Frame Number: 34
Acceptor (Background Subtraction) Frame Number: 35
Acceptor (Background Subtraction) Frame Number: 36
Acceptor (Background Subtraction) Frame Number: 37
Acceptor (Background Subtraction) Frame Number: 38
Acceptor (Background Subtraction) Frame Number: 39
Acceptor (Background Subtraction) Frame Number: 40
Acceptor (Background Subtraction) Frame Number: 41
Acceptor (Background Subtraction) Frame Number: 42
Acceptor (Background Subtraction) Frame Number: 43
Acceptor (Background Subtraction) Frame Number: 44
Acceptor (Background Subtraction) Frame Number: 45
Acceptor (Background Subtraction) Frame Number: 46
Acceptor (Background Subtraction) Frame Number: 47
Acceptor (Background Subtraction) Frame Number: 48
Acceptor (Background Subtraction) Frame Number: 49
Acceptor (Background Subtraction) Frame Number: 50
Acceptor (Background Subtraction) Frame Number: 51

Acceptor (Background Subtraction) Frame Number: 52
Acceptor (Background Subtraction) Frame Number: 53
Acceptor (Background Subtraction) Frame Number: 54
Acceptor (Background Subtraction) Frame Number: 55
Acceptor (Background Subtraction) Frame Number: 56
Acceptor (Background Subtraction) Frame Number: 57
Acceptor (Background Subtraction) Frame Number: 58
Acceptor (Background Subtraction) Frame Number: 59
Acceptor (Background Subtraction) Frame Number: 60
Acceptor (Background Subtraction) Frame Number: 61
Acceptor (Background Subtraction) Frame Number: 62
Acceptor (Background Subtraction) Frame Number: 63
Acceptor (Background Subtraction) Frame Number: 64
Acceptor (Background Subtraction) Frame Number: 65
Acceptor (Background Subtraction) Frame Number: 66
Acceptor (Background Subtraction) Frame Number: 67
Acceptor (Background Subtraction) Frame Number: 68
Acceptor (Background Subtraction) Frame Number: 69
Acceptor (Background Subtraction) Frame Number: 70
Acceptor (Background Subtraction) Frame Number: 71
Acceptor (Background Subtraction) Frame Number: 72
Acceptor (Background Subtraction) Frame Number: 73
Acceptor (Background Subtraction) Frame Number: 74
Acceptor (Background Subtraction) Frame Number: 75
Acceptor (Background Subtraction) Frame Number: 76
Acceptor (Background Subtraction) Frame Number: 77
Acceptor (Background Subtraction) Frame Number: 78
Acceptor (Background Subtraction) Frame Number: 79
Acceptor (Background Subtraction) Frame Number: 80
Acceptor (Background Subtraction) Frame Number: 81
Acceptor (Background Subtraction) Frame Number: 82
Acceptor (Background Subtraction) Frame Number: 83
Acceptor (Background Subtraction) Frame Number: 84
Acceptor (Background Subtraction) Frame Number: 85
Acceptor (Background Subtraction) Frame Number: 86
Acceptor (Background Subtraction) Frame Number: 87
Acceptor (Background Subtraction) Frame Number: 88
Acceptor (Background Subtraction) Frame Number: 89
Acceptor (Background Subtraction) Frame Number: 90
Acceptor (Background Subtraction) Frame Number: 91
Acceptor (Background Subtraction) Frame Number: 92
Acceptor (Background Subtraction) Frame Number: 93
Acceptor (Background Subtraction) Frame Number: 94
Acceptor (Background Subtraction) Frame Number: 95
Acceptor (Background Subtraction) Frame Number: 96
Acceptor (Background Subtraction) Frame Number: 97

```
Acceptor (Background Subtraction) Frame Number: 98
Acceptor (Background Subtraction) Frame Number: 99
Acceptor (Background Subtraction) Frame Number: 100
Acceptor (Background Subtraction) Frame Number: 101
Acceptor (Background Subtraction) Frame Number: 102
Acceptor (Background Subtraction) Frame Number: 103
Acceptor (Background Subtraction) Frame Number: 104
Acceptor (Background Subtraction) Frame Number: 105
Acceptor (Background Subtraction) Frame Number: 106
Acceptor (Background Subtraction) Frame Number: 107
Acceptor (Background Subtraction) Frame Number: 108
Acceptor (Background Subtraction) Frame Number: 109
Acceptor (Background Subtraction) Frame Number: 110
Acceptor (Background Subtraction) Frame Number: 111
Acceptor (Background Subtraction) Frame Number: 112
Acceptor (Background Subtraction) Frame Number: 113
Acceptor (Background Subtraction) Frame Number: 114
Acceptor (Background Subtraction) Frame Number: 115
Acceptor (Background Subtraction) Frame Number: 116
Acceptor (Background Subtraction) Frame Number: 117
Acceptor (Background Subtraction) Frame Number: 118
Acceptor (Background Subtraction) Frame Number: 119
Acceptor (Background Subtraction) Frame Number: 120
Acceptor (Background Subtraction) Frame Number: 121
Acceptor (Background Subtraction) Frame Number: 122
Acceptor (Background Subtraction) Frame Number: 123
Acceptor (Background Subtraction) Frame Number: 124
Acceptor (Background Subtraction) Time: 195 second(s)
Acceptor (Background Animation) Time: 97 second(s)
Saving Acceptor HDF5 stack in /Users/reviewer/fret-ibra/ibra/YC124/YC124.h5 [Time: 4
second(s)]
Saving Acceptor TIFF stack in /Users/reviewer/fret-
ibra/ibra/YC124/YC124_back_acceptor.tif [Time: 11 second(s)]
(py38) COMP:ibra reviewer$ ./ibra.py -c Config_YC124-1.cfg -a -t -s -v
/opt/anaconda3/envs/py38/lib/python3.8/site-packages/pims/api.py:191: UserWarning:
<class 'pims.tiff_stack.TiffStack_tifffile'> errored: 'NoneType' object has no attribute
'TiffFile'
  warn(message)
Donor (Background Subtraction) Frame Number: 1
Donor (Background Subtraction) Frame Number: 2
Donor (Background Subtraction) Frame Number: 3
Donor (Background Subtraction) Frame Number: 4
Donor (Background Subtraction) Frame Number: 5
Donor (Background Subtraction) Frame Number: 6
Donor (Background Subtraction) Frame Number: 7
Donor (Background Subtraction) Frame Number: 8
```

Donor (Background Subtraction) Frame Number: 9
Donor (Background Subtraction) Frame Number: 10
Donor (Background Subtraction) Frame Number: 11
Donor (Background Subtraction) Frame Number: 12
Donor (Background Subtraction) Frame Number: 13
Donor (Background Subtraction) Frame Number: 14
Donor (Background Subtraction) Frame Number: 15
Donor (Background Subtraction) Frame Number: 16
Donor (Background Subtraction) Frame Number: 17
Donor (Background Subtraction) Frame Number: 18
Donor (Background Subtraction) Frame Number: 19
Donor (Background Subtraction) Frame Number: 20
Donor (Background Subtraction) Frame Number: 21
Donor (Background Subtraction) Frame Number: 22
Donor (Background Subtraction) Frame Number: 23
Donor (Background Subtraction) Frame Number: 24
Donor (Background Subtraction) Frame Number: 25
Donor (Background Subtraction) Frame Number: 26
Donor (Background Subtraction) Frame Number: 27
Donor (Background Subtraction) Frame Number: 28
Donor (Background Subtraction) Frame Number: 29
Donor (Background Subtraction) Frame Number: 30
Donor (Background Subtraction) Frame Number: 31
Donor (Background Subtraction) Frame Number: 32
Donor (Background Subtraction) Frame Number: 33
Donor (Background Subtraction) Frame Number: 34
Donor (Background Subtraction) Frame Number: 35
Donor (Background Subtraction) Frame Number: 36
Donor (Background Subtraction) Frame Number: 37
Donor (Background Subtraction) Frame Number: 38
Donor (Background Subtraction) Frame Number: 39
Donor (Background Subtraction) Frame Number: 40
Donor (Background Subtraction) Frame Number: 41
Donor (Background Subtraction) Frame Number: 42
Donor (Background Subtraction) Frame Number: 43
Donor (Background Subtraction) Frame Number: 44
Donor (Background Subtraction) Frame Number: 45
Donor (Background Subtraction) Frame Number: 46
Donor (Background Subtraction) Frame Number: 47
Donor (Background Subtraction) Frame Number: 48
Donor (Background Subtraction) Frame Number: 49
Donor (Background Subtraction) Frame Number: 50
Donor (Background Subtraction) Frame Number: 51
Donor (Background Subtraction) Frame Number: 52
Donor (Background Subtraction) Frame Number: 53
Donor (Background Subtraction) Frame Number: 54

Donor (Background Subtraction) Frame Number: 55
Donor (Background Subtraction) Frame Number: 56
Donor (Background Subtraction) Frame Number: 57
Donor (Background Subtraction) Frame Number: 58
Donor (Background Subtraction) Frame Number: 59
Donor (Background Subtraction) Frame Number: 60
Donor (Background Subtraction) Frame Number: 61
Donor (Background Subtraction) Frame Number: 62
Donor (Background Subtraction) Frame Number: 63
Donor (Background Subtraction) Frame Number: 64
Donor (Background Subtraction) Frame Number: 65
Donor (Background Subtraction) Frame Number: 66
Donor (Background Subtraction) Frame Number: 67
Donor (Background Subtraction) Frame Number: 68
Donor (Background Subtraction) Frame Number: 69
Donor (Background Subtraction) Frame Number: 70
Donor (Background Subtraction) Frame Number: 71
Donor (Background Subtraction) Frame Number: 72
Donor (Background Subtraction) Frame Number: 73
Donor (Background Subtraction) Frame Number: 74
Donor (Background Subtraction) Frame Number: 75
Donor (Background Subtraction) Frame Number: 76
Donor (Background Subtraction) Frame Number: 77
Donor (Background Subtraction) Frame Number: 78
Donor (Background Subtraction) Frame Number: 79
Donor (Background Subtraction) Frame Number: 80
Donor (Background Subtraction) Frame Number: 81
Donor (Background Subtraction) Frame Number: 82
Donor (Background Subtraction) Frame Number: 83
Donor (Background Subtraction) Frame Number: 84
Donor (Background Subtraction) Frame Number: 85
Donor (Background Subtraction) Frame Number: 86
Donor (Background Subtraction) Frame Number: 87
Donor (Background Subtraction) Frame Number: 88
Donor (Background Subtraction) Frame Number: 89
Donor (Background Subtraction) Frame Number: 90
Donor (Background Subtraction) Frame Number: 91
Donor (Background Subtraction) Frame Number: 92
Donor (Background Subtraction) Frame Number: 93
Donor (Background Subtraction) Frame Number: 94
Donor (Background Subtraction) Frame Number: 95
Donor (Background Subtraction) Frame Number: 96
Donor (Background Subtraction) Frame Number: 97
Donor (Background Subtraction) Frame Number: 98
Donor (Background Subtraction) Frame Number: 99
Donor (Background Subtraction) Frame Number: 100

```
Donor (Background Subtraction) Frame Number: 101
Donor (Background Subtraction) Frame Number: 102
Donor (Background Subtraction) Frame Number: 103
Donor (Background Subtraction) Frame Number: 104
Donor (Background Subtraction) Frame Number: 105
Donor (Background Subtraction) Frame Number: 106
Donor (Background Subtraction) Frame Number: 107
Donor (Background Subtraction) Frame Number: 108
Donor (Background Subtraction) Frame Number: 109
Donor (Background Subtraction) Frame Number: 110
Donor (Background Subtraction) Frame Number: 111
Donor (Background Subtraction) Frame Number: 112
Donor (Background Subtraction) Frame Number: 113
Donor (Background Subtraction) Frame Number: 114
Donor (Background Subtraction) Frame Number: 115
Donor (Background Subtraction) Frame Number: 116
Donor (Background Subtraction) Frame Number: 117
Donor (Background Subtraction) Frame Number: 118
Donor (Background Subtraction) Frame Number: 119
Donor (Background Subtraction) Frame Number: 120
Donor (Background Subtraction) Frame Number: 121
Donor (Background Subtraction) Frame Number: 122
Donor (Background Subtraction) Frame Number: 123
Donor (Background Subtraction) Frame Number: 124
Donor (Background Subtraction) Time: 195 second(s)
Donor (Background Animation) Time: 91 second(s)
Saving Donor HDF5 stack in /Users/reviewer/fret-ibra/ibra/YC124/YC124.h5 [Time: 4
second(s)]
Saving Donor TIFF stack in /Users/reviewer/fret-ibra/ibra/YC124/YC124_back_donor.tif
[Time: 10 second(s)]
(py38) COMP:ibra reviewer$ ./ibra.py -c Config_YC124-2.cfg -a -t -s -v
(Ratio Processing) Frame Number: 1
(Ratio Processing) Frame Number: 2
(Ratio Processing) Frame Number: 3
(Ratio Processing) Frame Number: 4
(Ratio Processing) Frame Number: 5
(Ratio Processing) Frame Number: 6
(Ratio Processing) Frame Number: 7
(Ratio Processing) Frame Number: 8
(Ratio Processing) Frame Number: 9
(Ratio Processing) Frame Number: 10
(Ratio Processing) Frame Number: 11
(Ratio Processing) Frame Number: 12
(Ratio Processing) Frame Number: 13
(Ratio Processing) Frame Number: 14
(Ratio Processing) Frame Number: 15
```

(Ratio Processing) Frame Number: 16
(Ratio Processing) Frame Number: 17
(Ratio Processing) Frame Number: 18
(Ratio Processing) Frame Number: 19
(Ratio Processing) Frame Number: 20
(Ratio Processing) Frame Number: 21
(Ratio Processing) Frame Number: 22
(Ratio Processing) Frame Number: 23
(Ratio Processing) Frame Number: 24
(Ratio Processing) Frame Number: 25
(Ratio Processing) Frame Number: 26
(Ratio Processing) Frame Number: 27
(Ratio Processing) Frame Number: 28
(Ratio Processing) Frame Number: 29
(Ratio Processing) Frame Number: 30
(Ratio Processing) Frame Number: 31
(Ratio Processing) Frame Number: 32
(Ratio Processing) Frame Number: 33
(Ratio Processing) Frame Number: 34
(Ratio Processing) Frame Number: 35
(Ratio Processing) Frame Number: 36
(Ratio Processing) Frame Number: 37
(Ratio Processing) Frame Number: 38
(Ratio Processing) Frame Number: 39
(Ratio Processing) Frame Number: 40
(Ratio Processing) Frame Number: 41
(Ratio Processing) Frame Number: 42
(Ratio Processing) Frame Number: 43
(Ratio Processing) Frame Number: 44
(Ratio Processing) Frame Number: 45
(Ratio Processing) Frame Number: 46
(Ratio Processing) Frame Number: 47
(Ratio Processing) Frame Number: 48
(Ratio Processing) Frame Number: 49
(Ratio Processing) Frame Number: 50
(Ratio Processing) Frame Number: 51
(Ratio Processing) Frame Number: 52
(Ratio Processing) Frame Number: 53
(Ratio Processing) Frame Number: 54
(Ratio Processing) Frame Number: 55
(Ratio Processing) Frame Number: 56
(Ratio Processing) Frame Number: 57
(Ratio Processing) Frame Number: 58
(Ratio Processing) Frame Number: 59
(Ratio Processing) Frame Number: 60
(Ratio Processing) Frame Number: 61

(Ratio Processing) Frame Number: 62
(Ratio Processing) Frame Number: 63
(Ratio Processing) Frame Number: 64
(Ratio Processing) Frame Number: 65
(Ratio Processing) Frame Number: 66
(Ratio Processing) Frame Number: 67
(Ratio Processing) Frame Number: 68
(Ratio Processing) Frame Number: 69
(Ratio Processing) Frame Number: 70
(Ratio Processing) Frame Number: 71
(Ratio Processing) Frame Number: 72
(Ratio Processing) Frame Number: 73
(Ratio Processing) Frame Number: 74
(Ratio Processing) Frame Number: 75
(Ratio Processing) Frame Number: 76
(Ratio Processing) Frame Number: 77
(Ratio Processing) Frame Number: 78
(Ratio Processing) Frame Number: 79
(Ratio Processing) Frame Number: 80
(Ratio Processing) Frame Number: 81
(Ratio Processing) Frame Number: 82
(Ratio Processing) Frame Number: 83
(Ratio Processing) Frame Number: 84
(Ratio Processing) Frame Number: 85
(Ratio Processing) Frame Number: 86
(Ratio Processing) Frame Number: 87
(Ratio Processing) Frame Number: 88
(Ratio Processing) Frame Number: 89
(Ratio Processing) Frame Number: 90
(Ratio Processing) Frame Number: 91
(Ratio Processing) Frame Number: 92
(Ratio Processing) Frame Number: 93
(Ratio Processing) Frame Number: 94
(Ratio Processing) Frame Number: 95
(Ratio Processing) Frame Number: 96
(Ratio Processing) Frame Number: 97
(Ratio Processing) Frame Number: 98
(Ratio Processing) Frame Number: 99
(Ratio Processing) Frame Number: 100
(Ratio Processing) Frame Number: 101
(Ratio Processing) Frame Number: 102
(Ratio Processing) Frame Number: 103
(Ratio Processing) Frame Number: 104
(Ratio Processing) Frame Number: 105
(Ratio Processing) Frame Number: 106
(Ratio Processing) Frame Number: 107

```
(Ratio Processing) Frame Number: 108
(Ratio Processing) Frame Number: 109
(Ratio Processing) Frame Number: 110
(Ratio Processing) Frame Number: 111
(Ratio Processing) Frame Number: 112
(Ratio Processing) Frame Number: 113
(Ratio Processing) Frame Number: 114
(Ratio Processing) Frame Number: 115
(Ratio Processing) Frame Number: 116
(Ratio Processing) Frame Number: 117
(Ratio Processing) Frame Number: 118
(Ratio Processing) Frame Number: 119
(Ratio Processing) Frame Number: 120
(Ratio Processing) Frame Number: 121
(Ratio Processing) Frame Number: 122
(Ratio Processing) Frame Number: 123
(Ratio Processing) Frame Number: 124
(Ratio Processing) Time: 4 second(s)
Traceback (most recent call last):
  File "./ibra.py", line 180, in <module>
    main()
  File "./ibra.py", line 157, in main

rp.ratio(verbose,logger,work_out_path,crop,res,register,union,h5_save,tiff_save,frange)
  File "/Users/reviewer/fret-ibra/ibra/ratiometric_processing.py", line 266, in ratio
    ratio = ratio_calc(acceptorc,donorc)
  File "/Users/reviewer/fret-ibra/ibra/functions.py", line 323, in ratio_calc
    perc = np.percentile(ratio_flat[np.nonzero(ratio_flat)], [10, 90], interpolation='nearest')
  File "<__array_function__ internals>", line 5, in percentile
  File "/opt/anaconda3/envs/py38/lib/python3.8/site-
packages/numpy/lib/function_base.py", line 3705, in percentile
    return _quantile_unchecked(
  File "/opt/anaconda3/envs/py38/lib/python3.8/site-
packages/numpy/lib/function_base.py", line 3824, in _quantile_unchecked
    r, k = _ureduce(a, func=_quantile_ureduce_func, q=q, axis=axis, out=out,
  File "/opt/anaconda3/envs/py38/lib/python3.8/site-
packages/numpy/lib/function_base.py", line 3403, in _ureduce
    r = func(a, **kwargs)
  File "/opt/anaconda3/envs/py38/lib/python3.8/site-
packages/numpy/lib/function_base.py", line 3908, in _quantile_ureduce_func
    r = take(ap, indices, axis=axis, out=out)
  File "<__array_function__ internals>", line 5, in take
  File "/opt/anaconda3/envs/py38/lib/python3.8/site-
packages/numpy/core/fromnumeric.py", line 194, in take
    return _wrapfunc(a, 'take', indices, axis=axis, out=out, mode=mode)
```

```
  File "/opt/anaconda3/envs/py38/lib/python3.8/site-
packages/numpy/core/fromnumeric.py", line 61, in _wrapfunc
    return bound(*args, **kwds)
IndexError: cannot do a non-empty take from an empty axes.
```

YC170

```
(base) COMP:~ reviewer$ conda activate py38
(py38) COMP:~ reviewer$ cd fret-ibra/ibra/
(py38) COMP:ibra reviewer$ ./ibra.py -c Config_YC170-0.cfg -a -t -s -v
/opt/anaconda3/envs/py38/lib/python3.8/site-packages/pims/api.py:191: UserWarning:
<class 'pims.tiff_stack.TiffStack_tifffile'> errored: 'NoneType' object has no attribute
'TiffFile'
  warn(message)
Acceptor (Background Subtraction) Frame Number: 1
Acceptor (Background Subtraction) Frame Number: 2
Acceptor (Background Subtraction) Frame Number: 3
Acceptor (Background Subtraction) Frame Number: 4
Acceptor (Background Subtraction) Frame Number: 5
Acceptor (Background Subtraction) Frame Number: 6
Acceptor (Background Subtraction) Frame Number: 7
Acceptor (Background Subtraction) Frame Number: 8
Acceptor (Background Subtraction) Frame Number: 9
Acceptor (Background Subtraction) Frame Number: 10
Acceptor (Background Subtraction) Frame Number: 11
Acceptor (Background Subtraction) Frame Number: 12
Acceptor (Background Subtraction) Frame Number: 13
Acceptor (Background Subtraction) Frame Number: 14
Acceptor (Background Subtraction) Frame Number: 15
Acceptor (Background Subtraction) Frame Number: 16
Acceptor (Background Subtraction) Frame Number: 17
Acceptor (Background Subtraction) Frame Number: 18
Acceptor (Background Subtraction) Frame Number: 19
Acceptor (Background Subtraction) Frame Number: 20
Acceptor (Background Subtraction) Frame Number: 21
Acceptor (Background Subtraction) Frame Number: 22
Acceptor (Background Subtraction) Frame Number: 23
Acceptor (Background Subtraction) Frame Number: 24
Acceptor (Background Subtraction) Frame Number: 25
Acceptor (Background Subtraction) Frame Number: 26
Acceptor (Background Subtraction) Frame Number: 27
Acceptor (Background Subtraction) Frame Number: 28
Acceptor (Background Subtraction) Frame Number: 29
Acceptor (Background Subtraction) Frame Number: 30
Acceptor (Background Subtraction) Frame Number: 31
```

Acceptor (Background Subtraction) Frame Number: 32
Acceptor (Background Subtraction) Frame Number: 33
Acceptor (Background Subtraction) Frame Number: 34
Acceptor (Background Subtraction) Frame Number: 35
Acceptor (Background Subtraction) Frame Number: 36
Acceptor (Background Subtraction) Frame Number: 37
Acceptor (Background Subtraction) Frame Number: 38
Acceptor (Background Subtraction) Frame Number: 39
Acceptor (Background Subtraction) Frame Number: 40
Acceptor (Background Subtraction) Frame Number: 41
Acceptor (Background Subtraction) Frame Number: 42
Acceptor (Background Subtraction) Frame Number: 43
Acceptor (Background Subtraction) Frame Number: 44
Acceptor (Background Subtraction) Frame Number: 45
Acceptor (Background Subtraction) Frame Number: 46
Acceptor (Background Subtraction) Frame Number: 47
Acceptor (Background Subtraction) Frame Number: 48
Acceptor (Background Subtraction) Frame Number: 49
Acceptor (Background Subtraction) Frame Number: 50
Acceptor (Background Subtraction) Frame Number: 51
Acceptor (Background Subtraction) Frame Number: 52
Acceptor (Background Subtraction) Frame Number: 53
Acceptor (Background Subtraction) Frame Number: 54
Acceptor (Background Subtraction) Frame Number: 55
Acceptor (Background Subtraction) Frame Number: 56
Acceptor (Background Subtraction) Frame Number: 57
Acceptor (Background Subtraction) Frame Number: 58
Acceptor (Background Subtraction) Frame Number: 59
Acceptor (Background Subtraction) Frame Number: 60
Acceptor (Background Subtraction) Frame Number: 61
Acceptor (Background Subtraction) Frame Number: 62
Acceptor (Background Subtraction) Frame Number: 63
Acceptor (Background Subtraction) Frame Number: 64
Acceptor (Background Subtraction) Frame Number: 65
Acceptor (Background Subtraction) Frame Number: 66
Acceptor (Background Subtraction) Frame Number: 67
Acceptor (Background Subtraction) Frame Number: 68
Acceptor (Background Subtraction) Frame Number: 69
Acceptor (Background Subtraction) Frame Number: 70
Acceptor (Background Subtraction) Frame Number: 71
Acceptor (Background Subtraction) Frame Number: 72
Acceptor (Background Subtraction) Frame Number: 73
Acceptor (Background Subtraction) Frame Number: 74
Acceptor (Background Subtraction) Frame Number: 75
Acceptor (Background Subtraction) Frame Number: 76
Acceptor (Background Subtraction) Frame Number: 77

Acceptor (Background Subtraction) Frame Number: 78
Acceptor (Background Subtraction) Frame Number: 79
Acceptor (Background Subtraction) Frame Number: 80
Acceptor (Background Subtraction) Frame Number: 81
Acceptor (Background Subtraction) Frame Number: 82
Acceptor (Background Subtraction) Frame Number: 83
Acceptor (Background Subtraction) Frame Number: 84
Acceptor (Background Subtraction) Frame Number: 85
Acceptor (Background Subtraction) Frame Number: 86
Acceptor (Background Subtraction) Frame Number: 87
Acceptor (Background Subtraction) Frame Number: 88
Acceptor (Background Subtraction) Frame Number: 89
Acceptor (Background Subtraction) Frame Number: 90
Acceptor (Background Subtraction) Frame Number: 91
Acceptor (Background Subtraction) Frame Number: 92
Acceptor (Background Subtraction) Frame Number: 93
Acceptor (Background Subtraction) Frame Number: 94
Acceptor (Background Subtraction) Frame Number: 95
Acceptor (Background Subtraction) Frame Number: 96
Acceptor (Background Subtraction) Frame Number: 97
Acceptor (Background Subtraction) Frame Number: 98
Acceptor (Background Subtraction) Frame Number: 99
Acceptor (Background Subtraction) Frame Number: 100
Acceptor (Background Subtraction) Frame Number: 101
Acceptor (Background Subtraction) Frame Number: 102
Acceptor (Background Subtraction) Frame Number: 103
Acceptor (Background Subtraction) Frame Number: 104
Acceptor (Background Subtraction) Frame Number: 105
Acceptor (Background Subtraction) Frame Number: 106
Acceptor (Background Subtraction) Frame Number: 107
Acceptor (Background Subtraction) Frame Number: 108
Acceptor (Background Subtraction) Frame Number: 109
Acceptor (Background Subtraction) Frame Number: 110
Acceptor (Background Subtraction) Frame Number: 111
Acceptor (Background Subtraction) Frame Number: 112
Acceptor (Background Subtraction) Frame Number: 113
Acceptor (Background Subtraction) Frame Number: 114
Acceptor (Background Subtraction) Frame Number: 115
Acceptor (Background Subtraction) Frame Number: 116
Acceptor (Background Subtraction) Frame Number: 117
Acceptor (Background Subtraction) Frame Number: 118
Acceptor (Background Subtraction) Frame Number: 119
Acceptor (Background Subtraction) Frame Number: 120
Acceptor (Background Subtraction) Frame Number: 121
Acceptor (Background Subtraction) Frame Number: 122
Acceptor (Background Subtraction) Frame Number: 123

Acceptor (Background Subtraction) Frame Number: 124
Acceptor (Background Subtraction) Frame Number: 125
Acceptor (Background Subtraction) Frame Number: 126
Acceptor (Background Subtraction) Frame Number: 127
Acceptor (Background Subtraction) Frame Number: 128
Acceptor (Background Subtraction) Frame Number: 129
Acceptor (Background Subtraction) Frame Number: 130
Acceptor (Background Subtraction) Frame Number: 131
Acceptor (Background Subtraction) Frame Number: 132
Acceptor (Background Subtraction) Frame Number: 133
Acceptor (Background Subtraction) Frame Number: 134
Acceptor (Background Subtraction) Frame Number: 135
Acceptor (Background Subtraction) Frame Number: 136
Acceptor (Background Subtraction) Frame Number: 137
Acceptor (Background Subtraction) Frame Number: 138
Acceptor (Background Subtraction) Frame Number: 139
Acceptor (Background Subtraction) Frame Number: 140
Acceptor (Background Subtraction) Frame Number: 141
Acceptor (Background Subtraction) Frame Number: 142
Acceptor (Background Subtraction) Frame Number: 143
Acceptor (Background Subtraction) Frame Number: 144
Acceptor (Background Subtraction) Frame Number: 145
Acceptor (Background Subtraction) Frame Number: 146
Acceptor (Background Subtraction) Frame Number: 147
Acceptor (Background Subtraction) Frame Number: 148
Acceptor (Background Subtraction) Frame Number: 149
Acceptor (Background Subtraction) Frame Number: 150
Acceptor (Background Subtraction) Frame Number: 151
Acceptor (Background Subtraction) Frame Number: 152
Acceptor (Background Subtraction) Frame Number: 153
Acceptor (Background Subtraction) Frame Number: 154
Acceptor (Background Subtraction) Frame Number: 155
Acceptor (Background Subtraction) Frame Number: 156
Acceptor (Background Subtraction) Frame Number: 157
Acceptor (Background Subtraction) Frame Number: 158
Acceptor (Background Subtraction) Frame Number: 159
Acceptor (Background Subtraction) Frame Number: 160
Acceptor (Background Subtraction) Frame Number: 161
Acceptor (Background Subtraction) Frame Number: 162
Acceptor (Background Subtraction) Frame Number: 163
Acceptor (Background Subtraction) Frame Number: 164
Acceptor (Background Subtraction) Frame Number: 165
Acceptor (Background Subtraction) Frame Number: 166
Acceptor (Background Subtraction) Frame Number: 167
Acceptor (Background Subtraction) Frame Number: 168
Acceptor (Background Subtraction) Frame Number: 169

Acceptor (Background Subtraction) Frame Number: 170
Acceptor (Background Subtraction) Time: 190 second(s)
Acceptor (Background Animation) Time: 124 second(s)
Saving Acceptor HDF5 stack in /Users/reviewer/fret-ibra/ibra/YC170/YC170.h5 [Time: 3 second(s)]
Saving Acceptor TIFF stack in /Users/reviewer/fret-ibra/ibra/YC170/YC170_back_acceptor.tif [Time: 7 second(s)]
(py38) COMP:ibra reviewer$ ./ibra.py -c Config_YC170-1.cfg -a -t -s -v
/opt/anaconda3/envs/py38/lib/python3.8/site-packages/pims/api.py:191: UserWarning: <class 'pims.tiff_stack.TiffStack_tifffile'> errored: 'NoneType' object has no attribute 'TiffFile'
  warn(message)
Donor (Background Subtraction) Frame Number: 1
Donor (Background Subtraction) Frame Number: 2
Donor (Background Subtraction) Frame Number: 3
Donor (Background Subtraction) Frame Number: 4
Donor (Background Subtraction) Frame Number: 5
Donor (Background Subtraction) Frame Number: 6
Donor (Background Subtraction) Frame Number: 7
Donor (Background Subtraction) Frame Number: 8
Donor (Background Subtraction) Frame Number: 9
Donor (Background Subtraction) Frame Number: 10
Donor (Background Subtraction) Frame Number: 11
Donor (Background Subtraction) Frame Number: 12
Donor (Background Subtraction) Frame Number: 13
Donor (Background Subtraction) Frame Number: 14
Donor (Background Subtraction) Frame Number: 15
Donor (Background Subtraction) Frame Number: 16
Donor (Background Subtraction) Frame Number: 17
Donor (Background Subtraction) Frame Number: 18
Donor (Background Subtraction) Frame Number: 19
Donor (Background Subtraction) Frame Number: 20
Donor (Background Subtraction) Frame Number: 21
Donor (Background Subtraction) Frame Number: 22
Donor (Background Subtraction) Frame Number: 23
Donor (Background Subtraction) Frame Number: 24
Donor (Background Subtraction) Frame Number: 25
Donor (Background Subtraction) Frame Number: 26
Donor (Background Subtraction) Frame Number: 27
Donor (Background Subtraction) Frame Number: 28
Donor (Background Subtraction) Frame Number: 29
Donor (Background Subtraction) Frame Number: 30
Donor (Background Subtraction) Frame Number: 31
Donor (Background Subtraction) Frame Number: 32
Donor (Background Subtraction) Frame Number: 33
Donor (Background Subtraction) Frame Number: 34

Donor (Background Subtraction) Frame Number: 35
Donor (Background Subtraction) Frame Number: 36
Donor (Background Subtraction) Frame Number: 37
Donor (Background Subtraction) Frame Number: 38
Donor (Background Subtraction) Frame Number: 39
Donor (Background Subtraction) Frame Number: 40
Donor (Background Subtraction) Frame Number: 41
Donor (Background Subtraction) Frame Number: 42
Donor (Background Subtraction) Frame Number: 43
Donor (Background Subtraction) Frame Number: 44
Donor (Background Subtraction) Frame Number: 45
Donor (Background Subtraction) Frame Number: 46
Donor (Background Subtraction) Frame Number: 47
Donor (Background Subtraction) Frame Number: 48
Donor (Background Subtraction) Frame Number: 49
Donor (Background Subtraction) Frame Number: 50
Donor (Background Subtraction) Frame Number: 51
Donor (Background Subtraction) Frame Number: 52
Donor (Background Subtraction) Frame Number: 53
Donor (Background Subtraction) Frame Number: 54
Donor (Background Subtraction) Frame Number: 55
Donor (Background Subtraction) Frame Number: 56
Donor (Background Subtraction) Frame Number: 57
Donor (Background Subtraction) Frame Number: 58
Donor (Background Subtraction) Frame Number: 59
Donor (Background Subtraction) Frame Number: 60
Donor (Background Subtraction) Frame Number: 61
Donor (Background Subtraction) Frame Number: 62
Donor (Background Subtraction) Frame Number: 63
Donor (Background Subtraction) Frame Number: 64
Donor (Background Subtraction) Frame Number: 65
Donor (Background Subtraction) Frame Number: 66
Donor (Background Subtraction) Frame Number: 67
Donor (Background Subtraction) Frame Number: 68
Donor (Background Subtraction) Frame Number: 69
Donor (Background Subtraction) Frame Number: 70
Donor (Background Subtraction) Frame Number: 71
Donor (Background Subtraction) Frame Number: 72
Donor (Background Subtraction) Frame Number: 73
Donor (Background Subtraction) Frame Number: 74
Donor (Background Subtraction) Frame Number: 75
Donor (Background Subtraction) Frame Number: 76
Donor (Background Subtraction) Frame Number: 77
Donor (Background Subtraction) Frame Number: 78
Donor (Background Subtraction) Frame Number: 79
Donor (Background Subtraction) Frame Number: 80

Donor (Background Subtraction) Frame Number: 81
Donor (Background Subtraction) Frame Number: 82
Donor (Background Subtraction) Frame Number: 83
Donor (Background Subtraction) Frame Number: 84
Donor (Background Subtraction) Frame Number: 85
Donor (Background Subtraction) Frame Number: 86
Donor (Background Subtraction) Frame Number: 87
Donor (Background Subtraction) Frame Number: 88
Donor (Background Subtraction) Frame Number: 89
Donor (Background Subtraction) Frame Number: 90
Donor (Background Subtraction) Frame Number: 91
Donor (Background Subtraction) Frame Number: 92
Donor (Background Subtraction) Frame Number: 93
Donor (Background Subtraction) Frame Number: 94
Donor (Background Subtraction) Frame Number: 95
Donor (Background Subtraction) Frame Number: 96
Donor (Background Subtraction) Frame Number: 97
Donor (Background Subtraction) Frame Number: 98
Donor (Background Subtraction) Frame Number: 99
Donor (Background Subtraction) Frame Number: 100
Donor (Background Subtraction) Frame Number: 101
Donor (Background Subtraction) Frame Number: 102
Donor (Background Subtraction) Frame Number: 103
Donor (Background Subtraction) Frame Number: 104
Donor (Background Subtraction) Frame Number: 105
Donor (Background Subtraction) Frame Number: 106
Donor (Background Subtraction) Frame Number: 107
Donor (Background Subtraction) Frame Number: 108
Donor (Background Subtraction) Frame Number: 109
Donor (Background Subtraction) Frame Number: 110
Donor (Background Subtraction) Frame Number: 111
Donor (Background Subtraction) Frame Number: 112
Donor (Background Subtraction) Frame Number: 113
Donor (Background Subtraction) Frame Number: 114
Donor (Background Subtraction) Frame Number: 115
Donor (Background Subtraction) Frame Number: 116
Donor (Background Subtraction) Frame Number: 117
Donor (Background Subtraction) Frame Number: 118
Donor (Background Subtraction) Frame Number: 119
Donor (Background Subtraction) Frame Number: 120
Donor (Background Subtraction) Frame Number: 121
Donor (Background Subtraction) Frame Number: 122
Donor (Background Subtraction) Frame Number: 123
Donor (Background Subtraction) Frame Number: 124
Donor (Background Subtraction) Frame Number: 125
Donor (Background Subtraction) Frame Number: 126

Donor (Background Subtraction) Frame Number: 127
Donor (Background Subtraction) Frame Number: 128
Donor (Background Subtraction) Frame Number: 129
Donor (Background Subtraction) Frame Number: 130
Donor (Background Subtraction) Frame Number: 131
Donor (Background Subtraction) Frame Number: 132
Donor (Background Subtraction) Frame Number: 133
Donor (Background Subtraction) Frame Number: 134
Donor (Background Subtraction) Frame Number: 135
Donor (Background Subtraction) Frame Number: 136
Donor (Background Subtraction) Frame Number: 137
Donor (Background Subtraction) Frame Number: 138
Donor (Background Subtraction) Frame Number: 139
Donor (Background Subtraction) Frame Number: 140
Donor (Background Subtraction) Frame Number: 141
Donor (Background Subtraction) Frame Number: 142
Donor (Background Subtraction) Frame Number: 143
Donor (Background Subtraction) Frame Number: 144
Donor (Background Subtraction) Frame Number: 145
Donor (Background Subtraction) Frame Number: 146
Donor (Background Subtraction) Frame Number: 147
Donor (Background Subtraction) Frame Number: 148
Donor (Background Subtraction) Frame Number: 149
Donor (Background Subtraction) Frame Number: 150
Donor (Background Subtraction) Frame Number: 151
Donor (Background Subtraction) Frame Number: 152
Donor (Background Subtraction) Frame Number: 153
Donor (Background Subtraction) Frame Number: 154
Donor (Background Subtraction) Frame Number: 155
Donor (Background Subtraction) Frame Number: 156
Donor (Background Subtraction) Frame Number: 157
Donor (Background Subtraction) Frame Number: 158
Donor (Background Subtraction) Frame Number: 159
Donor (Background Subtraction) Frame Number: 160
Donor (Background Subtraction) Frame Number: 161
Donor (Background Subtraction) Frame Number: 162
Donor (Background Subtraction) Frame Number: 163
Donor (Background Subtraction) Frame Number: 164
Donor (Background Subtraction) Frame Number: 165
Donor (Background Subtraction) Frame Number: 166
Donor (Background Subtraction) Frame Number: 167
Donor (Background Subtraction) Frame Number: 168
Donor (Background Subtraction) Frame Number: 169
Donor (Background Subtraction) Frame Number: 170
Donor (Background Subtraction) Time: 197 second(s)
Donor (Background Animation) Time: 126 second(s)

```
Saving Donor HDF5 stack in /Users/reviewer/fret-ibra/ibra/YC170/YC170.h5 [Time: 4
second(s)]
Saving Donor TIFF stack in /Users/reviewer/fret-ibra/ibra/YC170/YC170_back_donor.tif
[Time: 7 second(s)]
(py38) COMP:ibra reviewer$ ./ibra.py -c Config_YC170-2.cfg -a -t -s -v
(Ratio Processing) Frame Number: 1
(Ratio Processing) Frame Number: 2
(Ratio Processing) Frame Number: 3
(Ratio Processing) Frame Number: 4
(Ratio Processing) Frame Number: 5
(Ratio Processing) Frame Number: 6
(Ratio Processing) Frame Number: 7
(Ratio Processing) Frame Number: 8
(Ratio Processing) Frame Number: 9
(Ratio Processing) Frame Number: 10
(Ratio Processing) Frame Number: 11
(Ratio Processing) Frame Number: 12
(Ratio Processing) Frame Number: 13
(Ratio Processing) Frame Number: 14
(Ratio Processing) Frame Number: 15
(Ratio Processing) Frame Number: 16
(Ratio Processing) Frame Number: 17
(Ratio Processing) Frame Number: 18
(Ratio Processing) Frame Number: 19
(Ratio Processing) Frame Number: 20
(Ratio Processing) Frame Number: 21
(Ratio Processing) Frame Number: 22
(Ratio Processing) Frame Number: 23
(Ratio Processing) Frame Number: 24
(Ratio Processing) Frame Number: 25
(Ratio Processing) Frame Number: 26
(Ratio Processing) Frame Number: 27
(Ratio Processing) Frame Number: 28
(Ratio Processing) Frame Number: 29
(Ratio Processing) Frame Number: 30
(Ratio Processing) Frame Number: 31
(Ratio Processing) Frame Number: 32
(Ratio Processing) Frame Number: 33
(Ratio Processing) Frame Number: 34
(Ratio Processing) Frame Number: 35
(Ratio Processing) Frame Number: 36
(Ratio Processing) Frame Number: 37
(Ratio Processing) Frame Number: 38
(Ratio Processing) Frame Number: 39
(Ratio Processing) Frame Number: 40
(Ratio Processing) Frame Number: 41
```

(Ratio Processing) Frame Number: 42
(Ratio Processing) Frame Number: 43
(Ratio Processing) Frame Number: 44
(Ratio Processing) Frame Number: 45
(Ratio Processing) Frame Number: 46
(Ratio Processing) Frame Number: 47
(Ratio Processing) Frame Number: 48
(Ratio Processing) Frame Number: 49
(Ratio Processing) Frame Number: 50
(Ratio Processing) Frame Number: 51
(Ratio Processing) Frame Number: 52
(Ratio Processing) Frame Number: 53
(Ratio Processing) Frame Number: 54
(Ratio Processing) Frame Number: 55
(Ratio Processing) Frame Number: 56
(Ratio Processing) Frame Number: 57
(Ratio Processing) Frame Number: 58
(Ratio Processing) Frame Number: 59
(Ratio Processing) Frame Number: 60
(Ratio Processing) Frame Number: 61
(Ratio Processing) Frame Number: 62
(Ratio Processing) Frame Number: 63
(Ratio Processing) Frame Number: 64
(Ratio Processing) Frame Number: 65
(Ratio Processing) Frame Number: 66
(Ratio Processing) Frame Number: 67
(Ratio Processing) Frame Number: 68
(Ratio Processing) Frame Number: 69
(Ratio Processing) Frame Number: 70
(Ratio Processing) Frame Number: 71
(Ratio Processing) Frame Number: 72
(Ratio Processing) Frame Number: 73
(Ratio Processing) Frame Number: 74
(Ratio Processing) Frame Number: 75
(Ratio Processing) Frame Number: 76
(Ratio Processing) Frame Number: 77
(Ratio Processing) Frame Number: 78
(Ratio Processing) Frame Number: 79
(Ratio Processing) Frame Number: 80
(Ratio Processing) Frame Number: 81
(Ratio Processing) Frame Number: 82
(Ratio Processing) Frame Number: 83
(Ratio Processing) Frame Number: 84
(Ratio Processing) Frame Number: 85
(Ratio Processing) Frame Number: 86
(Ratio Processing) Frame Number: 87

(Ratio Processing) Frame Number: 88
(Ratio Processing) Frame Number: 89
(Ratio Processing) Frame Number: 90
(Ratio Processing) Frame Number: 91
(Ratio Processing) Frame Number: 92
(Ratio Processing) Frame Number: 93
(Ratio Processing) Frame Number: 94
(Ratio Processing) Frame Number: 95
(Ratio Processing) Frame Number: 96
(Ratio Processing) Frame Number: 97
(Ratio Processing) Frame Number: 98
(Ratio Processing) Frame Number: 99
(Ratio Processing) Frame Number: 100
(Ratio Processing) Frame Number: 101
(Ratio Processing) Frame Number: 102
(Ratio Processing) Frame Number: 103
(Ratio Processing) Frame Number: 104
(Ratio Processing) Frame Number: 105
(Ratio Processing) Frame Number: 106
(Ratio Processing) Frame Number: 107
(Ratio Processing) Frame Number: 108
(Ratio Processing) Frame Number: 109
(Ratio Processing) Frame Number: 110
(Ratio Processing) Frame Number: 111
(Ratio Processing) Frame Number: 112
(Ratio Processing) Frame Number: 113
(Ratio Processing) Frame Number: 114
(Ratio Processing) Frame Number: 115
(Ratio Processing) Frame Number: 116
(Ratio Processing) Frame Number: 117
(Ratio Processing) Frame Number: 118
(Ratio Processing) Frame Number: 119
(Ratio Processing) Frame Number: 120
(Ratio Processing) Frame Number: 121
(Ratio Processing) Frame Number: 122
(Ratio Processing) Frame Number: 123
(Ratio Processing) Frame Number: 124
(Ratio Processing) Frame Number: 125
(Ratio Processing) Frame Number: 126
(Ratio Processing) Frame Number: 127
(Ratio Processing) Frame Number: 128
(Ratio Processing) Frame Number: 129
(Ratio Processing) Frame Number: 130
(Ratio Processing) Frame Number: 131
(Ratio Processing) Frame Number: 132
(Ratio Processing) Frame Number: 133

```
(Ratio Processing) Frame Number: 134
(Ratio Processing) Frame Number: 135
(Ratio Processing) Frame Number: 136
(Ratio Processing) Frame Number: 137
(Ratio Processing) Frame Number: 138
(Ratio Processing) Frame Number: 139
(Ratio Processing) Frame Number: 140
(Ratio Processing) Frame Number: 141
(Ratio Processing) Frame Number: 142
(Ratio Processing) Frame Number: 143
(Ratio Processing) Frame Number: 144
(Ratio Processing) Frame Number: 145
(Ratio Processing) Frame Number: 146
(Ratio Processing) Frame Number: 147
(Ratio Processing) Frame Number: 148
(Ratio Processing) Frame Number: 149
(Ratio Processing) Frame Number: 150
(Ratio Processing) Frame Number: 151
(Ratio Processing) Frame Number: 152
(Ratio Processing) Frame Number: 153
(Ratio Processing) Frame Number: 154
(Ratio Processing) Frame Number: 155
(Ratio Processing) Frame Number: 156
(Ratio Processing) Frame Number: 157
(Ratio Processing) Frame Number: 158
(Ratio Processing) Frame Number: 159
(Ratio Processing) Frame Number: 160
(Ratio Processing) Frame Number: 161
(Ratio Processing) Frame Number: 162
(Ratio Processing) Frame Number: 163
(Ratio Processing) Frame Number: 164
(Ratio Processing) Frame Number: 165
(Ratio Processing) Frame Number: 166
(Ratio Processing) Frame Number: 167
(Ratio Processing) Frame Number: 168
(Ratio Processing) Frame Number: 169
(Ratio Processing) Frame Number: 170
(Ratio Processing) Time: 4 second(s)
Traceback (most recent call last):
  File "./ibra.py", line 180, in <module>
    main()
  File "./ibra.py", line 157, in main

rp.ratio(verbose,logger,work_out_path,crop,res,register,union,h5_save,tiff_save,frange)
  File "/Users/reviewer/fret-ibra/ibra/ratiometric_processing.py", line 266, in ratio
    ratio = ratio_calc(acceptorc,donorc)
```

```
  File "/Users/reviewer/fret-ibra/ibra/functions.py", line 323, in ratio_calc
    perc = np.percentile(ratio_flat[np.nonzero(ratio_flat)], [10, 90], interpolation='nearest')
  File "<__array_function__ internals>", line 5, in percentile
  File "/opt/anaconda3/envs/py38/lib/python3.8/site-
packages/numpy/lib/function_base.py", line 3705, in percentile
    return _quantile_unchecked(
  File "/opt/anaconda3/envs/py38/lib/python3.8/site-
packages/numpy/lib/function_base.py", line 3824, in _quantile_unchecked
    r, k = _ureduce(a, func=_quantile_ureduce_func, q=q, axis=axis, out=out,
  File "/opt/anaconda3/envs/py38/lib/python3.8/site-
packages/numpy/lib/function_base.py", line 3403, in _ureduce
    r = func(a, **kwargs)
  File "/opt/anaconda3/envs/py38/lib/python3.8/site-
packages/numpy/lib/function_base.py", line 3908, in _quantile_ureduce_func
    r = take(ap, indices, axis=axis, out=out)
  File "<__array_function__ internals>", line 5, in take
  File "/opt/anaconda3/envs/py38/lib/python3.8/site-
packages/numpy/core/fromnumeric.py", line 194, in take
    return _wrapfunc(a, 'take', indices, axis=axis, out=out, mode=mode)
  File "/opt/anaconda3/envs/py38/lib/python3.8/site-
packages/numpy/core/fromnumeric.py", line 61, in _wrapfunc
    return bound(*args, **kwds)
IndexError: cannot do a non-empty take from an empty axes.
```
