# Supplementary material for: Fast and flexible processing of large FRET image stacks using the FRET-IBRA toolkit
Source: PLoS Comput Biol. 2022 Apr 4;18(4):e1009242. doi: 10.1371/journal.pcbi.1009242 (PMC9009774; doi:10.1371/journal.pcbi.1009242)
Supplement: Attachment — Submitted filename: Report_Details_PCOMPBIOL-D-21-01180R1.pdf [file pcbi.1009242.s003.pdf]

Report details PCOMPBIOL-D-21-01180R1

Figure of the Brachypodium roots example

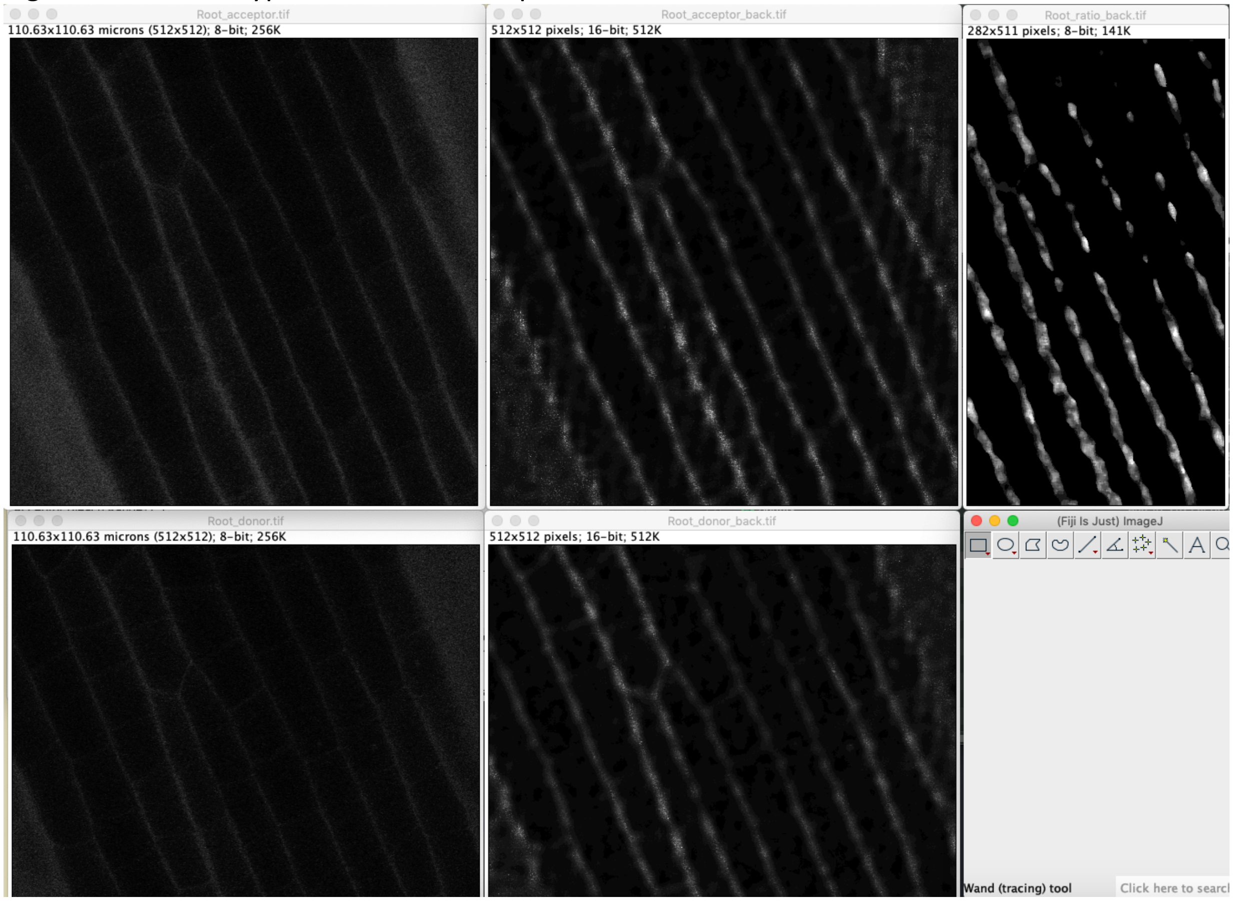

Figure of the Brachypodium roots example

Ratio processing log:

```
Acceptor (Background Subtraction) Frame Number: 1
Acceptor (Background Subtraction) Frame Number: 2
Acceptor (Background Subtraction) Frame Number: 3
Acceptor (Background Subtraction) Frame Number: 4
Acceptor (Background Subtraction) Frame Number: 5
Acceptor (Background Subtraction) Frame Number: 6
Acceptor (Background Subtraction) Frame Number: 7
Acceptor (Background Subtraction) Frame Number: 8
Acceptor (Background Subtraction) Frame Number: 9
Acceptor (Background Subtraction) Frame Number: 10
Acceptor (Background Subtraction) Frame Number: 11
Acceptor (Background Subtraction) Frame Number: 12
Acceptor (Background Subtraction) Frame Number: 13
Acceptor (Background Subtraction) Frame Number: 14
Acceptor (Background Subtraction) Frame Number: 15
Acceptor (Background Subtraction) Frame Number: 16
Acceptor (Background Subtraction) Frame Number: 17
Acceptor (Background Subtraction) Frame Number: 18
```

Acceptor (Background Subtraction) Frame Number: 19
Acceptor (Background Subtraction) Frame Number: 20
Acceptor (Background Subtraction) Frame Number: 21
Acceptor (Background Subtraction) Frame Number: 22
Acceptor (Background Subtraction) Frame Number: 23
Acceptor (Background Subtraction) Frame Number: 24
Acceptor (Background Subtraction) Frame Number: 25
Acceptor (Background Subtraction) Frame Number: 26
Acceptor (Background Subtraction) Frame Number: 27
Acceptor (Background Subtraction) Frame Number: 28
Acceptor (Background Subtraction) Frame Number: 29
Acceptor (Background Subtraction) Frame Number: 30
Acceptor (Background Subtraction) Frame Number: 31
Acceptor (Background Subtraction) Frame Number: 32
Acceptor (Background Subtraction) Frame Number: 33
Acceptor (Background Subtraction) Frame Number: 34
Acceptor (Background Subtraction) Frame Number: 35
Acceptor (Background Subtraction) Frame Number: 36
Acceptor (Background Subtraction) Frame Number: 37
Acceptor (Background Subtraction) Frame Number: 38
Acceptor (Background Subtraction) Frame Number: 39
Acceptor (Background Subtraction) Frame Number: 40
Acceptor (Background Subtraction) Frame Number: 41
Acceptor (Background Subtraction) Frame Number: 42
Acceptor (Background Subtraction) Frame Number: 43
Acceptor (Background Subtraction) Frame Number: 44
Acceptor (Background Subtraction) Frame Number: 45
Acceptor (Background Subtraction) Frame Number: 46
Acceptor (Background Subtraction) Frame Number: 47
Acceptor (Background Subtraction) Frame Number: 48
Acceptor (Background Subtraction) Frame Number: 49
Acceptor (Background Subtraction) Frame Number: 50
Acceptor (Background Subtraction) Frame Number: 51
Acceptor (Background Subtraction) Frame Number: 52
Acceptor (Background Subtraction) Frame Number: 53
Acceptor (Background Subtraction) Frame Number: 54
Acceptor (Background Subtraction) Frame Number: 55
Acceptor (Background Subtraction) Frame Number: 56
Acceptor (Background Subtraction) Frame Number: 57
Acceptor (Background Subtraction) Frame Number: 58
Acceptor (Background Subtraction) Frame Number: 59
Acceptor (Background Subtraction) Frame Number: 60
Acceptor (Background Subtraction) Frame Number: 61
Acceptor (Background Subtraction) Frame Number: 62
Acceptor (Background Subtraction) Frame Number: 63
Acceptor (Background Subtraction) Frame Number: 64
Acceptor (Background Subtraction) Frame Number: 65
Acceptor (Background Subtraction) Frame Number: 66
Acceptor (Background Subtraction) Frame Number: 67
Acceptor (Background Subtraction) Frame Number: 68
Acceptor (Background Subtraction) Frame Number: 69
Acceptor (Background Subtraction) Frame Number: 70
Acceptor (Background Subtraction) Frame Number: 71
Acceptor (Background Subtraction) Frame Number: 72
Acceptor (Background Subtraction) Frame Number: 73
Acceptor (Background Subtraction) Frame Number: 74
Acceptor (Background Subtraction) Frame Number: 75
Acceptor (Background Subtraction) Frame Number: 76
Acceptor (Background Subtraction) Frame Number: 77
Acceptor (Background Subtraction) Frame Number: 78
Acceptor (Background Subtraction) Frame Number: 79
Acceptor (Background Subtraction) Frame Number: 80
Acceptor (Background Subtraction) Frame Number: 81
Acceptor (Background Subtraction) Frame Number: 82
Acceptor (Background Subtraction) Frame Number: 83

```
Acceptor (Background Subtraction) Frame Number: 84
Acceptor (Background Subtraction) Frame Number: 85
Acceptor (Background Subtraction) Frame Number: 86
Acceptor (Background Subtraction) Frame Number: 87
Acceptor (Background Subtraction) Frame Number: 88
Acceptor (Background Subtraction) Frame Number: 89
Acceptor (Background Subtraction) Frame Number: 90
Acceptor (Background Subtraction) Frame Number: 91
Acceptor (Background Subtraction) Frame Number: 92
Acceptor (Background Subtraction) Frame Number: 93
Acceptor (Background Subtraction) Frame Number: 94
Acceptor (Background Subtraction) Frame Number: 95
Acceptor (Background Subtraction) Frame Number: 96
Acceptor (Background Subtraction) Frame Number: 97
Acceptor (Background Subtraction) Frame Number: 98
Acceptor (Background Subtraction) Frame Number: 99
Acceptor (Background Subtraction) Frame Number: 100
Acceptor (Background Subtraction) Frame Number: 101
Acceptor (Background Subtraction) Frame Number: 102
Acceptor (Background Subtraction) Frame Number: 103
Acceptor (Background Subtraction) Frame Number: 104
Acceptor (Background Subtraction) Frame Number: 105
Acceptor (Background Subtraction) Frame Number: 106
Acceptor (Background Subtraction) Frame Number: 107
Acceptor (Background Subtraction) Frame Number: 108
Acceptor (Background Subtraction) Frame Number: 109
Acceptor (Background Subtraction) Frame Number: 110
Acceptor (Background Subtraction) Frame Number: 111
Acceptor (Background Subtraction) Frame Number: 112
Acceptor (Background Subtraction) Frame Number: 113
Acceptor (Background Subtraction) Frame Number: 114
Acceptor (Background Subtraction) Frame Number: 115
Acceptor (Background Subtraction) Frame Number: 116
Acceptor (Background Subtraction) Frame Number: 117
Acceptor (Background Subtraction) Frame Number: 118
Acceptor (Background Subtraction) Frame Number: 119
Acceptor (Background Subtraction) Frame Number: 120
Acceptor (Background Subtraction) Frame Number: 121
Acceptor (Background Subtraction) Frame Number: 122
Acceptor (Background Subtraction) Frame Number: 123
Acceptor (Background Subtraction) Frame Number: 124
Acceptor (Background Subtraction) Time: 49 second(s)
Acceptor (Background Animation) Time: 79 second(s)
Saving Acceptor HDF5 stack in /Users/duser/fret-ibra/ibra/YC124/YC124.h5 [Time: 8
second(s)]
Saving Acceptor TIFF stack in /Users/duser/fret-ibra/ibra/YC124/YC124_back_acceptor.tif
[Time: 10 second(s)]
/opt/anaconda3/envs/py38/lib/python3.8/site-packages/pims/api.py:191: UserWarning: <class
'pims.tiff_stack.TiffStack_tifffile'> errored: 'NoneType' object has no attribute
'TiffFile'
  warn(message)
Donor (Background Subtraction) Frame Number: 1
Donor (Background Subtraction) Frame Number: 2
Donor (Background Subtraction) Frame Number: 3
Donor (Background Subtraction) Frame Number: 4
Donor (Background Subtraction) Frame Number: 5
Donor (Background Subtraction) Frame Number: 6
Donor (Background Subtraction) Frame Number: 7
Donor (Background Subtraction) Frame Number: 8
Donor (Background Subtraction) Frame Number: 9
Donor (Background Subtraction) Frame Number: 10
Donor (Background Subtraction) Frame Number: 11
Donor (Background Subtraction) Frame Number: 12
Donor (Background Subtraction) Frame Number: 13
Donor (Background Subtraction) Frame Number: 14
```

Donor (Background Subtraction) Frame Number: 15
Donor (Background Subtraction) Frame Number: 16
Donor (Background Subtraction) Frame Number: 17
Donor (Background Subtraction) Frame Number: 18
Donor (Background Subtraction) Frame Number: 19
Donor (Background Subtraction) Frame Number: 20
Donor (Background Subtraction) Frame Number: 21
Donor (Background Subtraction) Frame Number: 22
Donor (Background Subtraction) Frame Number: 23
Donor (Background Subtraction) Frame Number: 24
Donor (Background Subtraction) Frame Number: 25
Donor (Background Subtraction) Frame Number: 26
Donor (Background Subtraction) Frame Number: 27
Donor (Background Subtraction) Frame Number: 28
Donor (Background Subtraction) Frame Number: 29
Donor (Background Subtraction) Frame Number: 30
Donor (Background Subtraction) Frame Number: 31
Donor (Background Subtraction) Frame Number: 32
Donor (Background Subtraction) Frame Number: 33
Donor (Background Subtraction) Frame Number: 34
Donor (Background Subtraction) Frame Number: 35
Donor (Background Subtraction) Frame Number: 36
Donor (Background Subtraction) Frame Number: 37
Donor (Background Subtraction) Frame Number: 38
Donor (Background Subtraction) Frame Number: 39
Donor (Background Subtraction) Frame Number: 40
Donor (Background Subtraction) Frame Number: 41
Donor (Background Subtraction) Frame Number: 42
Donor (Background Subtraction) Frame Number: 43
Donor (Background Subtraction) Frame Number: 44
Donor (Background Subtraction) Frame Number: 45
Donor (Background Subtraction) Frame Number: 46
Donor (Background Subtraction) Frame Number: 47
Donor (Background Subtraction) Frame Number: 48
Donor (Background Subtraction) Frame Number: 49
Donor (Background Subtraction) Frame Number: 50
Donor (Background Subtraction) Frame Number: 51
Donor (Background Subtraction) Frame Number: 52
Donor (Background Subtraction) Frame Number: 53
Donor (Background Subtraction) Frame Number: 54
Donor (Background Subtraction) Frame Number: 55
Donor (Background Subtraction) Frame Number: 56
Donor (Background Subtraction) Frame Number: 57
Donor (Background Subtraction) Frame Number: 58
Donor (Background Subtraction) Frame Number: 59
Donor (Background Subtraction) Frame Number: 60
Donor (Background Subtraction) Frame Number: 61
Donor (Background Subtraction) Frame Number: 62
Donor (Background Subtraction) Frame Number: 63
Donor (Background Subtraction) Frame Number: 64
Donor (Background Subtraction) Frame Number: 65
Donor (Background Subtraction) Frame Number: 66
Donor (Background Subtraction) Frame Number: 67
Donor (Background Subtraction) Frame Number: 68
Donor (Background Subtraction) Frame Number: 69
Donor (Background Subtraction) Frame Number: 70
Donor (Background Subtraction) Frame Number: 71
Donor (Background Subtraction) Frame Number: 72
Donor (Background Subtraction) Frame Number: 73
Donor (Background Subtraction) Frame Number: 74
Donor (Background Subtraction) Frame Number: 75
Donor (Background Subtraction) Frame Number: 76
Donor (Background Subtraction) Frame Number: 77
Donor (Background Subtraction) Frame Number: 78
Donor (Background Subtraction) Frame Number: 79

```
Donor (Background Subtraction) Frame Number: 80
Donor (Background Subtraction) Frame Number: 81
Donor (Background Subtraction) Frame Number: 82
Donor (Background Subtraction) Frame Number: 83
Donor (Background Subtraction) Frame Number: 84
Donor (Background Subtraction) Frame Number: 85
Donor (Background Subtraction) Frame Number: 86
Donor (Background Subtraction) Frame Number: 87
Donor (Background Subtraction) Frame Number: 88
Donor (Background Subtraction) Frame Number: 89
Donor (Background Subtraction) Frame Number: 90
Donor (Background Subtraction) Frame Number: 91
Donor (Background Subtraction) Frame Number: 92
Donor (Background Subtraction) Frame Number: 93
Donor (Background Subtraction) Frame Number: 94
Donor (Background Subtraction) Frame Number: 95
Donor (Background Subtraction) Frame Number: 96
Donor (Background Subtraction) Frame Number: 97
Donor (Background Subtraction) Frame Number: 98
Donor (Background Subtraction) Frame Number: 99
Donor (Background Subtraction) Frame Number: 100
Donor (Background Subtraction) Frame Number: 101
Donor (Background Subtraction) Frame Number: 102
Donor (Background Subtraction) Frame Number: 103
Donor (Background Subtraction) Frame Number: 104
Donor (Background Subtraction) Frame Number: 105
Donor (Background Subtraction) Frame Number: 106
Donor (Background Subtraction) Frame Number: 107
Donor (Background Subtraction) Frame Number: 108
Donor (Background Subtraction) Frame Number: 109
Donor (Background Subtraction) Frame Number: 110
Donor (Background Subtraction) Frame Number: 111
Donor (Background Subtraction) Frame Number: 112
Donor (Background Subtraction) Frame Number: 113
Donor (Background Subtraction) Frame Number: 114
Donor (Background Subtraction) Frame Number: 115
Donor (Background Subtraction) Frame Number: 116
Donor (Background Subtraction) Frame Number: 117
Donor (Background Subtraction) Frame Number: 118
Donor (Background Subtraction) Frame Number: 119
Donor (Background Subtraction) Frame Number: 120
Donor (Background Subtraction) Frame Number: 121
Donor (Background Subtraction) Frame Number: 122
Donor (Background Subtraction) Frame Number: 123
Donor (Background Subtraction) Frame Number: 124
Donor (Background Subtraction) Time: 52 second(s)
Donor (Background Animation) Time: 83 second(s)
Saving Donor HDF5 stack in /Users/duser/fret-ibra/ibra/YC124/YC124.h5 [Time: 8 second(s)]
Saving Donor TIFF stack in /Users/duser/fret-ibra/ibra/YC124/YC124_back_donor.tif [Time: 10
second(s)]
(Ratio Processing) Frame Number: 1
(Ratio Processing) Frame Number: 2
(Ratio Processing) Frame Number: 3
(Ratio Processing) Frame Number: 4
(Ratio Processing) Frame Number: 5
(Ratio Processing) Frame Number: 6
(Ratio Processing) Frame Number: 7
(Ratio Processing) Frame Number: 8
(Ratio Processing) Frame Number: 9
(Ratio Processing) Frame Number: 10
(Ratio Processing) Frame Number: 11
(Ratio Processing) Frame Number: 12
(Ratio Processing) Frame Number: 13
(Ratio Processing) Frame Number: 14
(Ratio Processing) Frame Number: 15
```

```
(Ratio Processing) Frame Number: 16
(Ratio Processing) Frame Number: 17
(Ratio Processing) Frame Number: 18
(Ratio Processing) Frame Number: 19
(Ratio Processing) Frame Number: 20
(Ratio Processing) Frame Number: 21
(Ratio Processing) Frame Number: 22
(Ratio Processing) Frame Number: 23
(Ratio Processing) Frame Number: 24
(Ratio Processing) Frame Number: 25
(Ratio Processing) Frame Number: 26
(Ratio Processing) Frame Number: 27
(Ratio Processing) Frame Number: 28
(Ratio Processing) Frame Number: 29
(Ratio Processing) Frame Number: 30
(Ratio Processing) Frame Number: 31
(Ratio Processing) Frame Number: 32
(Ratio Processing) Frame Number: 33
(Ratio Processing) Frame Number: 34
(Ratio Processing) Frame Number: 35
(Ratio Processing) Frame Number: 36
(Ratio Processing) Frame Number: 37
(Ratio Processing) Frame Number: 38
(Ratio Processing) Frame Number: 39
(Ratio Processing) Frame Number: 40
(Ratio Processing) Frame Number: 41
(Ratio Processing) Frame Number: 42
(Ratio Processing) Frame Number: 43
(Ratio Processing) Frame Number: 44
(Ratio Processing) Frame Number: 45
(Ratio Processing) Frame Number: 46
(Ratio Processing) Frame Number: 47
(Ratio Processing) Frame Number: 48
(Ratio Processing) Frame Number: 49
(Ratio Processing) Frame Number: 50
(Ratio Processing) Frame Number: 51
(Ratio Processing) Frame Number: 52
(Ratio Processing) Frame Number: 53
(Ratio Processing) Frame Number: 54
(Ratio Processing) Frame Number: 55
(Ratio Processing) Frame Number: 56
(Ratio Processing) Frame Number: 57
(Ratio Processing) Frame Number: 58
(Ratio Processing) Frame Number: 59
(Ratio Processing) Frame Number: 60
(Ratio Processing) Frame Number: 61
(Ratio Processing) Frame Number: 62
(Ratio Processing) Frame Number: 63
(Ratio Processing) Frame Number: 64
(Ratio Processing) Frame Number: 65
(Ratio Processing) Frame Number: 66
(Ratio Processing) Frame Number: 67
(Ratio Processing) Frame Number: 68
(Ratio Processing) Frame Number: 69
(Ratio Processing) Frame Number: 70
(Ratio Processing) Frame Number: 71
(Ratio Processing) Frame Number: 72
(Ratio Processing) Frame Number: 73
(Ratio Processing) Frame Number: 74
(Ratio Processing) Frame Number: 75
(Ratio Processing) Frame Number: 76
(Ratio Processing) Frame Number: 77
(Ratio Processing) Frame Number: 78
(Ratio Processing) Frame Number: 79
(Ratio Processing) Frame Number: 80
```

```
(Ratio Processing) Frame Number: 81
(Ratio Processing) Frame Number: 82
(Ratio Processing) Frame Number: 83
(Ratio Processing) Frame Number: 84
(Ratio Processing) Frame Number: 85
(Ratio Processing) Frame Number: 86
(Ratio Processing) Frame Number: 87
(Ratio Processing) Frame Number: 88
(Ratio Processing) Frame Number: 89
(Ratio Processing) Frame Number: 90
(Ratio Processing) Frame Number: 91
(Ratio Processing) Frame Number: 92
(Ratio Processing) Frame Number: 93
(Ratio Processing) Frame Number: 94
(Ratio Processing) Frame Number: 95
(Ratio Processing) Frame Number: 96
(Ratio Processing) Frame Number: 97
(Ratio Processing) Frame Number: 98
(Ratio Processing) Frame Number: 99
(Ratio Processing) Frame Number: 100
(Ratio Processing) Frame Number: 101
(Ratio Processing) Frame Number: 102
(Ratio Processing) Frame Number: 103
(Ratio Processing) Frame Number: 104
(Ratio Processing) Frame Number: 105
(Ratio Processing) Frame Number: 106
(Ratio Processing) Frame Number: 107
(Ratio Processing) Frame Number: 108
(Ratio Processing) Frame Number: 109
(Ratio Processing) Frame Number: 110
(Ratio Processing) Frame Number: 111
(Ratio Processing) Frame Number: 112
(Ratio Processing) Frame Number: 113
(Ratio Processing) Frame Number: 114
(Ratio Processing) Frame Number: 115
(Ratio Processing) Frame Number: 116
(Ratio Processing) Frame Number: 117
(Ratio Processing) Frame Number: 118
(Ratio Processing) Frame Number: 119
(Ratio Processing) Frame Number: 120
(Ratio Processing) Frame Number: 121
(Ratio Processing) Frame Number: 122
(Ratio Processing) Frame Number: 123
(Ratio Processing) Frame Number: 124
(Ratio Processing) Time: 23 second(s)
Exception in Tkinter callback
Traceback (most recent call last):
  File "/opt/anaconda3/envs/py38/lib/python3.8/site-packages/h5py/_hl/files.py", line 185,
in make_fid
    fid = h5f.open(name, h5f.ACC_RDWR, fapl=fapl)
  File "h5py/_objects.pyx", line 54, in h5py._objects.with_phil.wrapper
  File "h5py/_objects.pyx", line 55, in h5py._objects.with_phil.wrapper
  File "h5py/h5f.pyx", line 88, in h5py.h5f.open
OSError: Unable to open file (file is already open for read-only)

During handling of the above exception, another exception occurred:

Traceback (most recent call last):
  File "/opt/anaconda3/envs/py38/lib/python3.8/tkinter/__init__.py", line 1883, in __call__
    return self.func(*args)
  File "/Users/duser/fret-ibra/ibra/gui.py", line 343, in <lambda>
    ttk.Button(frm17, text="Run",
command=lambda:get_variables(dire,con_str,acc_str,don_str,range_str,res_str,par_state,nwind
ow_str\
  File "/Users/duser/fret-ibra/ibra/gui.py", line 99, in get_variables
```

```
    parameter_extraction.main_extract(cfname,bool(tiff_state.get()),verbose,bool(h5_state.g
et()),bool(anim_state.get()))
  File "/Users/duser/fret-ibra/ibra/parameter_extraction.py", line 107, in main_extract
    rp.ratio(verbose, logger, work_out_path, crop, res, register, union, h5_save,
tiff_save, frange)
  File "/Users/duser/fret-ibra/ibra/ratiometric_processing.py", line 270, in ratio
    time_evolution(acceptori,donori,work_out_path,'_intensity_nonbleach.png','Median
Intensity/Bit Depth',h5_save)
  File "/Users/duser/fret-ibra/ibra/functions.py", line 238, in time_evolution
    f = h5py.File(work_out_path+'_ratio_back.h5', 'a')
  File "/opt/anaconda3/envs/py38/lib/python3.8/site-packages/h5py/_hl/files.py", line 406,
in __init__
    fid = make_fid(name, mode, userblock_size,
  File "/opt/anaconda3/envs/py38/lib/python3.8/site-packages/h5py/_hl/files.py", line 187,
in make_fid
    fid = h5f.create(name, h5f.ACC_EXCL, fapl=fapl, fcpl=fcpl)
  File "h5py/_objects.pyx", line 54, in h5py._objects.with_phil.wrapper
  File "h5py/_objects.pyx", line 55, in h5py._objects.with_phil.wrapper
  File "h5py/h5f.pyx", line 108, in h5py.h5f.create
OSError: Unable to create file (file exists)
```
