# Supplementary material for: Fast and flexible processing of large FRET image stacks using the FRET-IBRA toolkit
Source: PLoS Comput Biol. 2022 Apr 4;18(4):e1009242. doi: 10.1371/journal.pcbi.1009242 (PMC9009774; doi:10.1371/journal.pcbi.1009242)
Supplement: Attachment — Submitted filename: FRET-IBRA_response_to_reviewers.docx [file pcbi.1009242.s002.docx]

# Detailed Response to the Reviewers’ Comments

First, we would like to thank the reviewers for their insightful and constructive criticisms. We have tried to address all the points they made in the revised version of our manuscript. Below you can find a point-by-point response to the reviewers’ comments. For ease of reading, the reviewers’ comments are shown in blue and our responses in italics.

## Reviewer #1

The data analysis pipeline presented in “Fast and flexible processing of large FRET image stacks using the FRET-IBRA toolkit” tackles major issues in obtaining robust quantitative estimates from fluorescence microscopy time series, especially when dealing with ratiometric reporters. The authors employ solid image processing techniques that can: a) subtract the background for each frame even with spatial variation; b) perform photobleaching correction; c) output a scaled ratio image that could be used for further quantitative analysis. This open-source tool has a clear and flexible methodology built on freely available and well-written Python code, which should appeal directly to the research of single cell growth/migration.

FRET-IBRA has the potential to become the go-to tool for researchers in the pollen tube and root hair community analyzing ratiometric data, which I believe would be enhanced if the authors address issues on three fronts: 1) benchmark; 2) automation; 3) distribution/interface. Although FRET-IBRA seems to offer a superior output than other related tools, this comparison was made with a single illustrative example and could benefit from directly quantifying variables of biological interest across multiple data-sets. Furthermore, automating the choice of certain parameters (mainly epsilon) could offer more objective criteria and minimize manual intervention. Lastly, despite providing a clear code and installation instructions in GitHub, the current distribution and interface requires a moderate level of programming experience that limits the potential users.

Regardless of the potential shortcomings, FRET-IBRA is certainly a useful tool that I personally intend to incorporate in my analyses. Below I further discuss points that can, hopefully, increase the adoption of this pipeline.

MAJOR POINTS

1) Benchmark

The authors use an example dataset to illustrate the workflow of FRET-IBRA and to compare it with other tools. While the representative dataset did provide a clear explanation of the method and its performance, it is difficult to evaluate how generalizable are the results when dealing with different datasets. Does the difference in performance with other tools hold up with different fluorescent reporters, microscope settings, image and object sizes? Relying on manual choice of parameters further complicates this matter (see discussion on automation), which is not to say that a manual option is not welcome. Furthermore, since the aim is to provide robust quantitative imaging, ideally one should also compare estimates of quantities-of-interest such as tip fluorescence ratio and tip-to-shank gradient. Showing the potential of FRET-IBRA to provide new biological insights from reliable spatiotemporal quantification of ratiometric fluorescence would greatly increase its impact within the aims of PLOS Computational Biology.

Reviewer #1 raises some important points here. Indeed, we tested FRET-IBRA with a variety of biosensors and fluorescent dyes, ratiometric or not. Neither the choice of the fluorophore nor the microscope system, as long as we used epifluorescence, had an effect on performance. However, confocal images often needed smoothing, for example by applying a Gaussian blur filter, before running FRET-IBRA. We have made a note to that effect in the tutorial on GitHub.

As for the estimation of quantities of interest, we fully agree with the Reviewer #1. To this end, we are developing additional software that uses the background subtracted images from FRET-IBRA as an input for in-depth ratio analysis, including the generation of localized regions of interest for tip and shank that allow detailed gradient analysis. While this software will be most useful for the analysis of tip-growing cells such as pollen tubes and root hairs, FRET-IBRA has much broader applications.

We have added several images and movies together with the associated log files to the FRET-IBRA GitHub repository. They feature different fluorophores and were taken with different microscopes and imaging setups. With this we hope to convince Reviewer #1 of the robustness of FRET-IBRA.

2) Automation

Although the possibility of correcting individual frames may be interesting when discontinuous events occur, requiring visual inspection and manual parameter choices compromises objectivity and increases the time required to analyze each series. How could one ensure comparable results when drastically different parameters are used to process different frames, channels and series?

I encourage the authors to provide an automatic choice of epsilon (and maybe even window size), which even if just “guesstimates” may help users to tune them as little as possible. Perhaps a few simplifying assumptions can aid in that endeavor, for example assuming that the number and location of foreground and background tiles should be very similar (if not identical) between channels.

Finally, while I fully appreciate that some experiments may lead to outliers, overcorrecting for them may lead to greater artifacts than simply disregarding specific time points. On that note, I would like to understand more the reasoning behind using image registration between acceptor and donor channels. Unless both channels are sampled with a large time interval, it seems to me that they should be nearly or completely identical. Thus, allowing for image registration – even if only a rigid linear transformation – seems to increase the chance of introducing artifacts. Although the online tutorial does mention that registration should be used only after choosing an “optimal” value of epsilon, there is seemingly no objective criteria for that. I can picture scenarios where an experimental intervention may displace the cell, where registration would be important to match its position, however this would not be between channels but between consecutive frames. I ask the authors to consider adding further explanation or a discussion on the matter.

Epsilon is highly dependent on the image itself, so even though we tested various automated methods to estimate epsilon, we found that they were inadequate in providing an acceptable epsilon value. Therefore, we just set a default value of 0.01 in the GUI/Config file based on past image stacks that we’ve tested, which we found to be an adequate starting point for the user.

The number of windows parameter (nwindows) has also been set to 40 by default. While this parameter will not work for all image resolutions, the returned error message has been updated to provide a suggested nwindow size based on the resolution.

FRET-IBRA was designed to fit seamlessly into other image processing pipelines that perform temporal analysis for quantities of interest such as tip velocity and tip-to-shank gradient over time. We noticed that deleting instead of correcting (or potentially, overcorrecting) individual frames resulted in outputs from which temporal analysis of any kind became unnecessarily complicated, particularly when derivatives of the quantities of interest are desired. This was our justification for programming FRET-IBRA in such a way as to allow the manual correction of a frame or a range of frames without a loss in performance.

Furthermore, epsilon is a clustering parameter whose behaviour is completely non-linear with respect to the amount of total fluorescent signal captured from a background subtracted image. Therefore, the epsilon value of outlier frames usually needs to be tweaked very slightly in order to dramatically improve the quality of the background subtraction process, resulting in a corrected frame with a very similar fluorescent signal profile to neighboring non-outlier frames.

In longer image stacks, we noticed that an inability to tackle temporally decaying fluorescent signals with a choice of epsilon made analysis extremely tedious. If there is a true discontinuity in the image stack (like sudden external lighting changes) as mentioned by Reviewer #1, this will have to be dealt with separately.

We added an image registration option for users who use a dual-view camera setup, where the two channels are captured simultaneously on two separate sensors. In our experience, even the most careful calibration of sensors does not guarantee perfect alignment of the two channels, resulting in artifacts in the ratio image. We added some text to make this option clearer for users.

3) Distribution/interface

The choice of implementing the FRET-IBRA pipeline in Python has multiple benefits but also imposes challenges to users without a moderate programming experience. Even for initiates, some of the requirements of the pipeline are notoriously cumbersome to install. Despite the clear GitHub instructions, in my personal experience the installation took considerable time and effort. Even after managing the installation, there were errors in the ratiometric module and in processing 1024x1024 images that I am unsure whether they stem from issues in the code or simply installation flaws (see attached report). Two of the datasets tested yielded apparently great results in terms of background subtraction in both channels, however the ratiometric module yielded the error: “IndexError: cannot do a non-empty take from an empty axes” (see report). The other two datasets with 1024x1024 images were not processed due to a “ValueError: cannot reshape array of size 1048576 into shape (40,25,newaxis,25)” (see report).

Regardless of my personal experience testing the pipeline, a few solutions would definitely attract more users. The most inclusive option would be a web-interface where the user could interactively tune parameters. A Python-based graphical user interface could also help attract users, but it would likely still require cumbersome installations. An interactive Jupyter notebook can be a great choice but still may require a little programming skill. I encourage the authors to consider what is their audience and what would be the friendliest (and yet achievable) solution.

Finally, if sufficient automation is achieved, it would be interesting to have the complete workflow performed sequentially for a given series with a single command (background subtraction for each series, ratiometric calculation and optional photobleaching correction). Ideally a “batch mode” could ensure that a complete data cohort is analyzed with comparable parameters, although it may still be desirable to maintain a level of intervention in specific frames.

Firstly, we would like to apologize to Reviewer #1 for the easily avoidable errors that were received. After some analysis, we found that both issues stemmed from a nwindow value that was unsuitable for the background subtraction algorithm to function. We’ve updated our error handling module to clearly highlight when the nwindow parameter is unsuitable along with a new suggested value.

Based on Reviewer #1’s suggestions and the given time constraints, we decided to create a simple python-based GUI that replicates the parameters present in the config file to promote ease of use. Existing config files can also be uploaded to the GUI from where they can be run directly. The tutorial on GitHub has been updated to explain how the GUI can be run.

We decided against using Jupyter notebooks as FRET-IBRA was meant to be an application that is optimized to either run standalone or as part of a larger end-to-end pipeline, hence its object-oriented design. Furthermore, Jupyter would require the installation of the same backend python packages without the ease of use of a simple GUI.

Furthermore, as suggested by Reviewer #1, we have added an option to run an “almost” complete workflow solution with a single click, which includes background subtraction for both channels using the **same** given epsilon value along with ratiometric processing. Photobleaching correction is hugely dependent on the range of frames and chosen fit based on the visual results of the ratiometric processing module, and was hence left out of the automated workflow.

MINOR POINTS

- Lacking a discussion on how correcting photobleaching can insert artifacts.

We thank Reviewer #1 for raising this point and introduced the following text in the discussion:

“It should be noted that bleaching correction must be used with caution as it can cause artifacts, such as masking of true signal intensity variations or overcorrection, resulting in a false increase in signal intensity if the detrending methods provided are not appropriate. Therefore, it may sometimes be advisable to accept overall attenuation of the resulting ratio signal in order to detect short-term fluctuations. Alternatively, bleaching correction can be performed using other detrending algorithms outside of FRET-IBRA.”

- The standard deviation can be arbitrarily reduced with the amount of smoothing used (Pg. 5 ln 112-113). This maybe the reason why we see a decrease in the standard deviation and maximum values with window size in table 1. Thus, it maybe not be completely fair to use the standard deviation to compare with methods that do not perform smoothing.

Reviewer #1 is right. We removed the standard deviation from the comparison table.

- Pg. 6 ln 132 “rendering any quantitative analysis impossible” is a strong assertion, I recommend softening it.

This suggestion has been implemented. “Rendering any quantitative analysis impossible” has been replaced by “rendering any quantitative analysis very difficult”.

- Pg. 6 ln 146-147 “true dynamics of the signal intensity” is a strong assertion, I recommend substituting “true” by “hidden” or other equivalent term.

This suggestion has been implemented. “True” has been replaced by “hidden”.

- Pg. 7 ln 150 “best possible signal-to-noise ratio” is a strong assertion, I recommend softening it.

This suggestion has been implemented. “Best possible signal-to-noise ratio” has been replaced by “robust signal-to-noise ratio”.

## Reviewer #2

Summary:

The paper proposes a processing tool for FRET image stacks, including i) background subtraction for acceptor and donor time-lapse images, ii) registration between acceptor and donor movies to produce ratio images and iii) photobleaching correction. It can be used to quantify the spatial distribution of calcium ions during pollen tube growth under mechanical constraints and the code is released under BSD license.

Generally, it is not a methodologically innovative paper, as the primary methods involved in the tool are based on existing methods. For example, background subtraction is based on DBSCAN clustering proposed in another paper. Registration between acceptor and donor movies are a standard rigid-body registration. Nevertheless, a tool that comprises multiple processing steps could be still useful in practice is if its usability and robustness are sufficiently demonstrated. Hence, I would suggest authors should consider evaluating their tool more thoroughly, use multiple movies, best captured by different microscopy under different conditions, or in multiple labs, to demonstrate the generalisability of the proposed tool. These issues should be addressed in the revision.

Major issues:

i) The background subtraction module is based on a DBSCAN clustering algorithm on image tiles to separate foreground and background, proposed in Schwarzfisher et al (2011). Since this clustering needs to be done for each frame in the movie and is generally slow, does the proposed tool improve the original algorithm in terms of speed?

The core clustering algorithm used in FRET-IBRA is still DBSCAN (runtime: O[n logn]) as proposed by Schwarzfisher et al (2011); however, the feature space has been modified to improve the quality of the foreground and background tile classification. The main speed improvements for background subtraction lie in the optimized implementation of the pre/postprocessing steps on the image stack as well as the inclusion of a parallel option. Since the DBSCAN algorithm runs completely independently on each frame, the parallel option allows for individual frames to be run on independent threads resulting in a (close to) linear improvement in speed with the number of threads used.

ii) Comparison between BaSiC: as far as I know, BaSiC has a function to correct background (by setting Fiji plugin “Temporal_drift of baseline” to be “replace with zero” and it has a zero-like background by then. So it is surprising that your BaSiC corrected movie still has a high background value. Nevertheless, BaSiC might have a problem with highly-correlated foreground, which seems to be the case of the present example (exemplary movie in the github example) and may not function properly (as that breaks the basic assumption of BaSiC)

From our experience, BaSiC is a fantastic tool for retrospective background subtraction that applies to quite a diverse range of imaging modalities, and was actually the first choice for the analysis of our pollen tube growth time lapse movies. However, after consultation with the author (who assisted us with the parameter settings), we reached the conclusion that the highly-correlated foreground that usually exists in single-cell growth imaging breaks the basic assumption of BaSiC, as suspected by Reviewer #2. Based on this feedback, we proceeded to create FRET-IBRA based on the algorithm from Schwarzfischer et al, which we found to work best for image stacks with a low number of moving objects.

iii) How many movies are in the evaluation set. It seems to me only one pair of movies, i.e. acceptor and donor, is used. In my opinion, that is not sufficient to evaluate the robustness of the tool, which is also my main criticism of the paper.

We have added several images and movies together with the associated log files to the FRET-IBRA GitHub repository. They feature different fluorophores and were taken with different microscope and imaging setups. With this we hope to convince Reviewer #2 of the robustness of FRET-IBRA.

Suggestions

i) If authors can also provide some biological-relevant down-stream analysis, they would be a more solid demonstration of the necessity of such a tool.

We developed FRET-IBRA to analyze large ratio imaging stacks of up to 3000 frames with the aim to study calcium dynamics in growing pollen tubes at a high spatial and temporal resolution. As this is ongoing work, we are not able to share in-depth analyses at this point. During our long but unsuccessful search for appropriate available tools, we discussed this issue with many people facing similar problems. Therefore, we believe that FRET-IBRA meets a need and have decided to make it available as soon as possible.

ii) Authors should also discuss the potential usage and limitation of the tool: when it works and when it could fail, to prevent misuse of the tool.

This suggestion has been implemented. The following text has been added to the manuscript:

“FRET-IBRA is built to process images with few objects of interest that are densely packed, allowing for accurate background estimation. As the background subtraction algorithm classifies tiles solely as foreground or background, images with a wide spatially homogenous distribution of cells or other objects of interest might not be processed correctly due to poor estimation of the background.”
