# Supplementary material for: Fast and flexible processing of large FRET image stacks using the FRET-IBRA toolkit
Source: PLoS Comput Biol. 2022 Apr 4;18(4):e1009242. doi: 10.1371/journal.pcbi.1009242 (PMC9009774; doi:10.1371/journal.pcbi.1009242)
Supplement: Attachment — Submitted filename: Munglani_revision2_response.docx [file pcbi.1009242.s004.docx]

# Detailed Response to the Reviewer’s Comments

We would like to thank Reviewer#1 for his helpful inputs, which made us aware of a few glitches in the code and led to a further improvement of the software. Upon request we added a longer ratiometric movie (YC_3_ sub) to the examples on GitHub. Due to the large size of the original stack (1713 frames), we only show the results from the analysis of a 100-frame substack as well as the signal intensity curves of the whole movie before and after bleaching correction. The full analysis of the whole movie has been made available to Reviewer#1. Below you can find a point-by-point response to the comments of Reviewer#1. For ease of reading, the reviewers’ comments are shown in blue and our responses in italics.

## Reviewer #1: OVERALL REMARKS

Munglani et al. addressed several points raised by the reviewers and presented: 1) a friendlier interface to the FRET-IBRA pipeline both in terms of user input (GUI) and workflow (complete background subtraction on both channels and ratiometric processing); 2) more examples of its biological applicability in the GitHub repository and 3) improved presentation/discussion of the results. These changes enhance the usability of the tool, although there are still a few shortcomings in the presentation and discussion of the results pointed out by both reviewers in the previous considerations. In my opinion, the major limitation of the manuscript still is to provide a clear demonstration of the biological applicability of the pipeline, which should be easily achievable by including long ratiometric movies and time series. I suggest illustrating the potential of FRET-IBRA in reporting the known oscillations in cytosolic calcium concentrations at the pollen tube tip. I reckon such addition to the manuscript should be easily achievable with data from the authors themselves, otherwise I would be happy to provide a suitable data set.

## DETAILED REMARKS

I am glad the authors took the effort to extend FRET-IBRA’s interface by providing a GUI and the option to do background subtraction for both channels and calculate the ratio, these changes should facilitate the accessibility of the pipeline for many users. Furthermore, the biological examples in the GitHub repository suggest a wide range of applicability of the tool although the data was not explicitly incorporated in the manuscript. Thus:

- Discussions about the examples in GitHub are limited since details are lacking

We have added README files to the respective examples describing the details of the underlying original images.

- Examples are single time points making it hard to evaluate the potential of the pipeline to generate a long ratiometric movie

The goal of adding these examples to the GitHub repository is to show that background subtraction and ratio image production works. However, during the test phase of FRET-IBRA, we have produced many long movies, some of which with more than 1000 frames. We added an example time lapse movie with 100 frames to GitHub and provided Reviewer#1 with the full length movie, including all the resulting files from the analysis with FRET-IBRA.

- The example in Brachypodium roots suggest strong spatial artifacts introduced by the background subtraction, especially of the acceptor channel (see figure in the attached report)

The Brachypodium roots example shows the limitations of the algorithm to automatically produce ratio images from images with low signal. In the case of an 8 bit image, manual ratio processing may lead to success. Background subtraction, however, works still well, even with very low signals. For 16 bit images with very low signals (≤ 4096), it usually helps to set the resolution parameter to 12 (see also answer to the first “minor point”).

- It is often difficult to interpret the biological relevance of the ratiometric output (e.g. Brachypodium roots example) without a clear reference, whereas studying its temporal behavior can provide greater reliability to the result

Reviewer#1 is right. As mentioned before, the Brachypodium roots example was chosen to demonstrate the robustness of the background subtraction algorithm also in a multicellular context. However, FRET-IBRA was indeed developed to analyse the temporal behaviour of ratiometric levels in (very) large image stacks of growing pollen tubes.

Regardless of other examples, the model system already presented in the manuscript (i.e. pollen tubes) is ideal to show whether the ratiometric processing can produce biologically meaningful results. Pollen tube growth is accompanied by notorious tip-focused oscillations in cytosolic calcium, which could be used as a proof-of-principle that the method can adequately capture biological oscillations.

We agree with Reviewer#1. We added an example (YC_3_sub) of a growing pollen tube that shows some oscillation.

## MINOR POINTS

- I am still unable to produce ratiometric stacks using 16 bit images, even when using the suggested nwindow (or others I tried). Admittedly this can be some issue on my end or something extremely simple to fix (check log in the attached report)

We would like to thank Reviewer#1 for bringing this error to our attention. After some examination, we found that a small typo introduced a casting error into the code which prevented some 16 bit images from being processed properly. This bug has now been fixed. The reviewer sent us four 16 bit stacks, which caused an error when he analysed them with FRET-IBRA. After correcting the code, image processing worked without problems. However, two of the stacks have very low pixel intensities for a 16 bit image (in the range of ~1000). For best results, we used 12 bit for the resolution in FRET-IBRA.

- I suggest adding an option to use bg/fg tiles from one channel to the other when image registration is not being used. This makes sense given the pixelwise stoichiometry assumption and would cut processing time nearly in half while avoiding artifacts due to different bg/fg definition in acceptor and donor channels. I emphasize that this is not a mandatory suggestion.

We thank Reviewer#1 for this suggestion. We explored this idea when first creating FRET-IBRA, but since most of the images we tested required registration, we did not implement it. Unfortunately, the way the code is currently structured and the image metadata is stored, this particular option would require significant time and effort to implement. We will consider implementing this option in a later release, however we believe that for the current release, FRET-IBRA has a relatively fast runtime and does not require this optimization.

- One idea is to include loess as a method for photobleaching correction, depending on the series length a sufficiently large span parameter should capture the slow trend. One idea is to use degree 1 only to avoid inserting artifacts in the trend. I emphasize that this is not a mandatory suggestion.

According to the valuable input from Reviewer#1, we added the loess fit with degree 1 as an option to the bleach correction module.

- Sentence in the abstract stating that the package has been “extensively used in quantifying the spatial distribution of calcium ions during pollen tube growth under mechanical constraints.” has not been discussed in the text and no direct data was shown. As it is formulated it sounds that FRET-IBRA has been extensively used in the community rather than on a particular data set. Please reformulate (e.g. specify the lab) or remove.

We reformulated the sentence.

- Ln 11. “cleanest signal possible” I recommend softening the assertion.

We reformulated the sentence.

- Ln 145. “without any artifacts” I suggest replacing by something like “without clear artifacts or extreme outliers”

We reformulated the sentence.
